# Materials Innovation and the Changing Face of Photocatalytic and Electrocatalytic Carbon Dioxide Reduction Research: From Metal Nanoclusters to Extended Frameworks

**DOI:** 10.1002/anie.202515667

**Published:** 2025-09-25

**Authors:** Tsukasa Irie, Kohki Sasaki, Saikat Das, Yuichi Negishi

**Affiliations:** ^1^ Institute of Multidisciplinary Research for Advanced Materials Tohoku University 2‐1‐1 Katahira Aoba‐ku Sendai 980‐8577 Japan

**Keywords:** Atomically precise nanoclusters, Carbon dioxide reduction, Electrocatalytic CO_2_ conversion, Photocatalytic nanomaterials, Porous framework catalysts

## Abstract

The accelerating rise in atmospheric CO_2_ levels, driven by anthropogenic emissions, underscores the urgent need for transformative carbon management strategies. Photocatalytic and electrocatalytic CO_2_ reduction reactions (CO_2_RRs) offer a promising route to valorize CO_2_ into energy‐rich fuels and chemical feedstocks, yet the intrinsic thermodynamic and kinetic barriers necessitate catalysts that combine high activity, selectivity, and durability. Recent breakthroughs in materials chemistry have spotlighted two emerging material classes—atomically precise metal nanoclusters (NCs) and extended reticular frameworks such as metal–organic frameworks (MOFs) and covalent organic frameworks (COFs)—as frontiers for next‐generation CO_2_RR catalysts. Both systems offer unparalleled opportunities to engineer active sites at the atomic and molecular levels, enabling precise modulation of local environments, electronic structures, and substrate interactions. This review brings these two materials paradigms under a common vision of precision‐controlled catalysis, highlighting how innovations in cluster size, framework topology, and chemical functionality dictate product selectivity and C─C coupling behavior. We discuss emerging mechanistic insights, synthetic strategies, and structure–function relationships and outline challenges in conductivity, stability, and scalability. Finally, we propose integrated design principles to guide the development of hybrid platforms for efficient and selective CO_2_ valorization.

## Graphical abstract

Innovative catalyst design is advancing precision‐controlled catalysis for CO_2_ reduction. This minireview investigates the forefront of photocatalytic and electrocatalytic CO_2_ reduction (CO_2_RR) leveraging metal nanoclusters (NCs), metal–organic frameworks (MOFs), and covalent organic frameworks (COFs), concentrating on structure–activity correlations, precision synthesis methods, and mechanistic elucidation of CO_2_ conversion pathways.

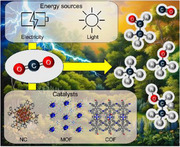

## 1 Introduction

Energy defines the arc of human civilization, driving economic growth, fostering social progress, and inspiring technological innovation. Yet, despite a growing recognition of environmental imperatives, modern society remains deeply anchored in fossil fuels, namely coal, oil, and natural gas, that continue to dominate the global energy landscape. This reliance drives an ever‐increasing accumulation of greenhouse gases in the atmosphere, with carbon dioxide (CO_2_) standing as the most consequential. The human‐induced surge in atmospheric CO_2_ serves as the linchpin of accelerating climate disruption, which manifests through global warming, sea level rise, increased ocean acidity, and heightened climatic volatility. While a phase‐out of fossil fuels is unrealistic in the near term, the gradual reorientation toward cleaner, sustainable energy systems is essential. Reimagining CO_2_ as a feedstock rather than waste is central to carbon‐neutral technologies.^[^ ^1^, ^2^, ^3^, ^4^ ^]^ Photocatalytic and electrocatalytic CO_2_ reduction (CO_2_RR) has emerged as a promising route, converting CO_2_ into diverse products—including CO, HCOOH, CH_4_, CH_3_OH, and C_2_ species—driven by solar or electrical energy.^[^ ^5^, ^6^, ^7^, ^8^, ^9^, ^10^ ^]^ Compared with thermocatalysis or bioenzymatic routes, these approaches offer mild operating conditions, greater stability, and compatibility with renewable energy, positioning them as scalable solutions.

However, CO_2_ is a highly stable, linear molecule with a strong C═O bond dissociation energy of ∼750 kJ mol^−1^ and significant thermodynamic inertness, rendering its reduction a demanding process.^[^ ^11^, ^12^ ^]^ Compounding this difficulty, CO_2_RR often contends with the hydrogen evolution reaction (HER), which proceeds more readily in aqueous media, thereby compromising selectivity and Faradaic efficiency. The activation of CO_2_ generally involves the formation of a CO_2_^•−^ intermediate, whose stabilization on catalyst surfaces is crucial for guiding the reaction pathway. Achieving product selectivity remains a fundamental challenge, particularly in steering the reaction toward multicarbon (C_2+_) products over simpler one‐carbon (C_1_) compounds. Recent developments in photocatalytic CO_2_RR have largely centered on the generation of C_1_ products (e.g., CO, HCOOH, CH_4_), with limited reports on C─C coupling and multicarbon outputs. In comparison, electrocatalytic CO_2_RR has made notable strides in the production of C_2_, C_3_, and even C_4_ products, primarily driven by copper‐based catalysts known for their unique ability to facilitate C─C bond formation.^[^ ^13^ ^]^ While copper has emerged as the benchmark for C_2+_ electrosynthesis,^[^ ^14^ ^]^ expanding the catalytic toolbox to include non‐Cu materials with comparable or superior selectivity and activity is an ongoing and strategically important research direction.^[^ ^15^ ^]^

**Figure 1:**
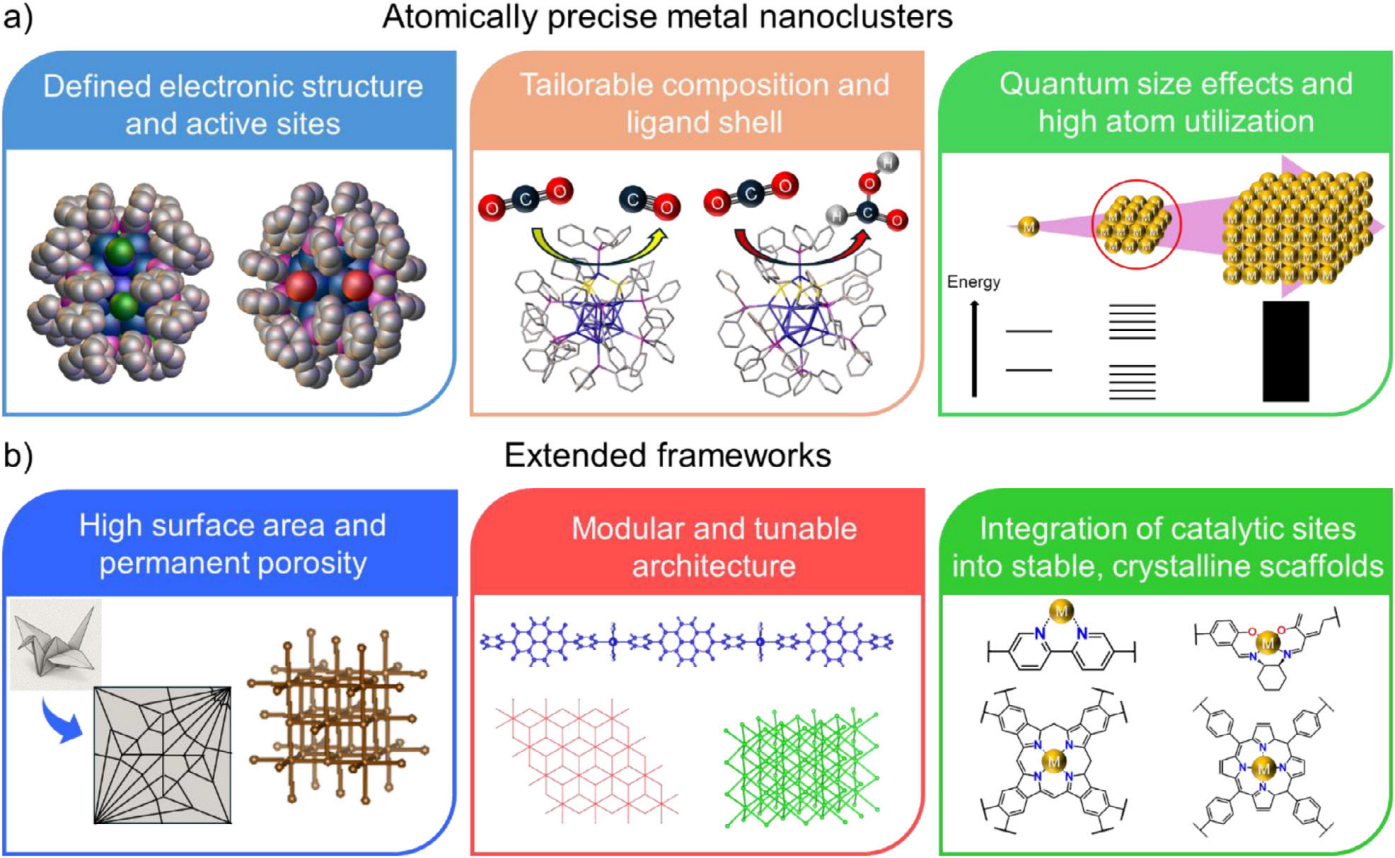
Distinctive merits of a) atomically precise metal nanoclusters and b) extended frameworks in facilitating CO_2_ photo/electroreduction.

Central to progress in CO_2_ reduction is the ability to craft catalysts with exacting control at the atomic and molecular scale, where both structure and electronic configuration can be finely tuned. Two material classes exemplifying this precision are metal nanoclusters (NCs)^[^ ^16^ ^]^ and reticular materials, namely metal–organic frameworks (MOFs) and covalent organic frameworks (COFs).^[^ ^17^ ^]^ Atomically precise metal NCs, typically composed of a few to tens of atoms, occupy the size regime between atoms and nanoparticles, distinguished by discrete energy levels, notable quantum confinement effects, and a rich presence of low‐coordinated surface atoms.^[^ ^18^, ^19^, ^20^, ^21^, ^22^, ^23^, ^24^, ^25^ ^]^ These unique characteristics offer unparalleled control over active site structure at the atomic level, enabling fundamental insights into catalytic mechanisms and precise tuning of electronic states for high selectivity and activity in CO_2_RR.^[^ ^26^, ^27^ ^]^ Their tunable size, composition, and ligand environment can influence product distribution and catalytic efficiency. Nevertheless, persistent issues such as limited structural resilience under operating conditions, aggregation tendencies, and difficulty in anchoring on supports remain critical bottlenecks to their long‐term performance. On the other hand, extended porous frameworks such as MOFs^[^ ^28^, ^29^, ^30^, ^31^, ^32^, ^33^, ^34^, ^35^, ^36^, ^37^ ^]^ and COFs^[^ ^38^, ^39^, ^40^, ^41^, ^42^, ^43^, ^44^, ^45^, ^46^, ^47^, ^48^, ^49^, ^50^, ^51^, ^52^ ^]^ offer a periodic and porous architecture capable of hosting active catalytic sites within a long‐range ordered network. Their large surface area, tailorable pore size, and modular design enable the incorporation of functional linkers, photosensitizers, or metal nodes that can participate directly in CO_2_ activation. MOFs, with their tunable metal nodes and versatile coordination environments, offer abundant and accessible active sites for catalytic activation, while COFs, built from robust covalent linkages and π‐conjugated architectures, excel in light harvesting and charge transport—together positioning both as promising and platforms for photo/electrocatalytic CO_2_RR. These materials excel at stabilizing intermediates, promoting charge separation, and facilitating selective reaction pathways. Yet, issues such as low conductivity (especially for pristine COFs), framework degradation under catalytic conditions, and challenges in achieving multicarbon products underscore the need for further structural innovation. The key advantageous properties unique to (a) atomically precise metal nanoclusters and (b) extended frameworks as photo‐ and electrocatalysts for CO_2_ reduction are schematically illustrated in Figure 1. Although metal NCs and reticular frameworks arise from distinct structural principles—discrete clusters versus extended periodic lattices—they share a common conceptual foundation in precision‐controlled catalysis. Both systems allow for atomic‐level customization of active sites, tunable electronic environments, and rational design strategies, enabling a deeper understanding of structure–function relationships in CO_2_ reduction catalysis. This convergence offers an exciting opportunity to unify disparate materials paradigms under a cohesive framework for advancing catalytic science.

In this review, we bring these two material families together to explore their potential and roles in addressing key challenges in CO_2_RR. Section 2 outlines the foundational aspects and techniques involved in CO_2_ activation and reduction, encompassing thermodynamic and kinetic considerations, key performance metrics such as overpotential, Faradaic efficiency, selectivity, and turnover frequency, and the analytical techniques used for product identification and mechanistic elucidation. Section 3 presents recent developments in metal NC‐based systems, emphasizing synthetic methodologies, structural design, and catalytic performance, with a particular focus on the influence of cluster size, electronic structure, and ligand environment on reaction pathways. Section 4 examines MOF‐ and COF‐based catalysts, highlighting how framework design, functionalization, and hybridization approaches enhance selectivity, stability, and multicarbon product formation. Finally, Section 5 identifies critical knowledge gaps and prevailing limitations and proposes future research directions, including strategies to promote C─C coupling, stabilize reactive intermediates, integrate photo‐ and electrocatalytic platforms, and design robust, scalable catalytic systems.

## 2 Principles and Techniques of Photo‐ and Electrocatalytic CO_2_ Reduction

A fundamental understanding of the underlying principles, mechanistic pathways, and performance indicators is crucial to designing efficient photocatalytic and electrocatalytic systems. This section offers a succinct overview of these principles, reviews common system designs, and discusses the analytical methods used to investigate catalytic activity and probe reaction mechanisms.

### 2.1 Fundamentals of CO_2_ Activation and Reduction

CO_2_ is a linear and thermodynamically stable molecule with a standard Gibbs free energy of formation (Δ*G*_f_°) of −394.4 kJ mol^−1^ and a highly negative first reduction potential (E° ≈ −1.90 V versus SHE in aqueous solution for the CO_2_/CO_2_^•−^ couple), making its activation and reduction inherently challenging.^[^ ^53^, ^54^ ^]^ Effective activation of CO_2_ necessitates breaking its symmetry and stabilizing reactive intermediates such as CO_2_^•−^, COOH*, HCOO*, or CHO*. Thermodynamic feasibility and kinetic accessibility are determined by:

- **Overpotential (η)**: The extra potential required beyond the thermodynamic value to drive the reaction at a practical rate.
- **Faradaic Efficiency (FE)**: The proportion of electrons that contribute to the desired product.
- **Selectivity**: The ability to produce a specific target molecule among a mixture of possible products (e.g., CO, CH_4_, C_2_H_4_).
- **Turnover Frequency (TOF)**: The number of product molecules generated per active site per unit time.

Understanding these metrics is critical for benchmarking catalysts under standardized conditions.

### 2.2 Photocatalytic CO_2_ Reduction: Artificial Photosystem Designs

**Figure 2:**
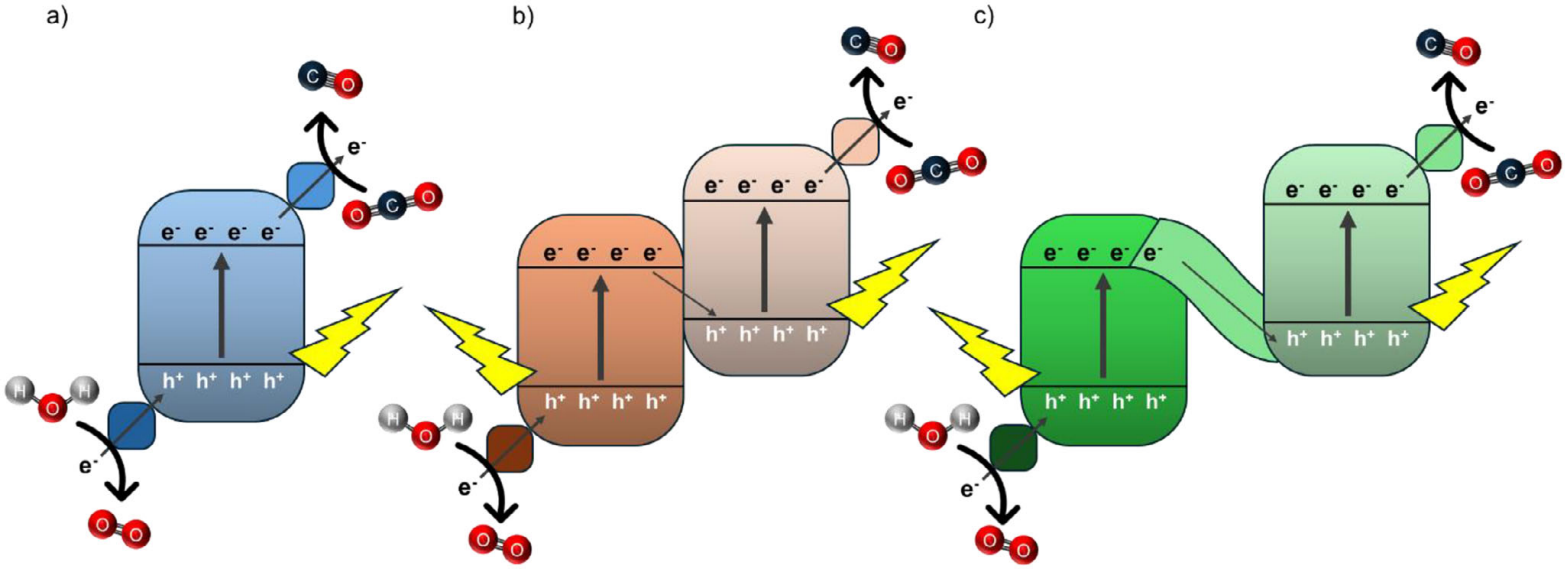
Schematic representations of photocatalytic systems: a) single excitation system employing a wide bandgap photocatalyst, b) multiple excitation system with photocatalysts connected via physical contact (heterojunctions), and c) Z‐scheme configuration with redox mediators facilitating charge transfer.

Photocatalytic CO_2_ reduction mimics natural photosynthesis harnessing solar energy to produce chemical fuels. Central to this process is the generation of electron‐hole pairs upon light absorption, followed by charge separation and transfer to drive the redox transformation of CO_2_. The design of artificial photocatalytic systems is commonly classified into the following architectures^[^ ^55^ ^]^:

- *Single Excitation System with Wide Bandgap Photocatalyst*. These systems employ a single semiconductor (e.g., TiO_2_, SrTiO_3_, g‐C_3_N_4_) that absorbs ultraviolet or near‐visible light to excite electrons from the valence band to the conduction band. The photoexcited electrons participate in CO_2_ reduction, while the photogenerated holes drive the oxidation of water or sacrificial agents (Figure 2a). Advantages include simple configuration, good stability, and ease of scale‐up. Drawbacks involve limited visible‐light response due to wide bandgap and inefficient charge separation.
- *Multiple Excitation System with Photocatalysts Connected* via *Physical Contact (Heterojunctions)*. This strategy involves the integration of two semiconductors with staggered band alignments (e.g., CdS/TiO_2_, BiVO_4_/g‐C_3_N_4_) to facilitate spatial separation of electrons and holes. Excited electrons and holes migrate across the interface to their respective active sites, improving charge separation and extending light absorption range (Figure 2b). While the benefits encompass superior charge carrier dynamics and broader solar spectrum utilization, issues like interfacial charge recombination and instability under reaction conditions remain significant hurdles.
- *Multiple Excitation System Connected by Electron Mediators (Z‐scheme)*. Inspired by the natural photosystem, the Z‐scheme couples two narrow bandgap semiconductors through a redox mediator (e.g., I^−^/IO_3_^−^, Fe^3+^/Fe^2+^) or solid‐state conductor. Each semiconductor absorbs light independently and performs either oxidation or reduction. The mediator enables electron transfer between the two semiconductors, maintaining charge balance and sustaining a significant redox potential difference across the system (Figure 2c). Key strengths include high redox capability, modular design, and diverse material combinations. Challenges involve energy losses from mediator diffusion or side reactions, as well as complex system optimization requirements.

### 2.3 Electrocatalytic CO_2_ Reduction: Electrolyzer Configurations and Mechanism

**Figure 3:**
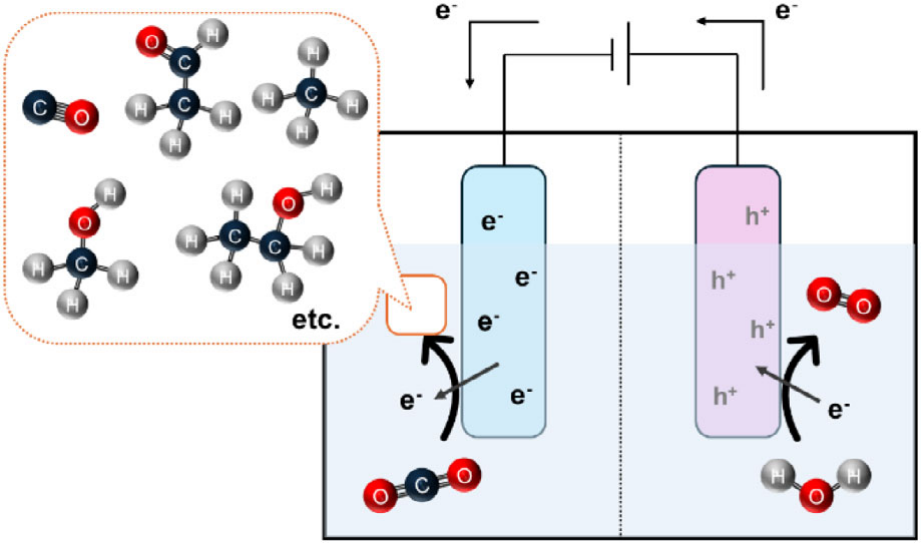
Schematic layout of an electrolyzer designed for CO_2_ electroreduction.

In electrocatalytic CO_2_RR, electrical energy is used to drive the reduction of CO_2_ at the cathode, while oxidation (typically water to O_2_) occurs at the anode (Figure 3). A standard CO_2_ electrolyzer consists of^[^ ^56^ ^]^:

- **Cathode**: Catalyst‐coated electrode for CO_2_ reduction.
- **Anode**: Oxygen evolution catalyst (e.g., IrO_2_, RuO_2_).
- **Electrolyte**: Aqueous or non‐aqueous medium that supports ion transport.
- **Membrane**: Separates cathodic and anodic compartments (e.g., proton exchange membrane or bipolar membrane).

**Process Overview**:

1. **CO_2_ supply**: CO_2_ gas is dissolved or fed directly to the catalyst layer.
2. **Electron transfer**: External voltage drives electrons to the cathode for CO_2_ reduction.
3. **Proton availability**: Protons (H^+^) or water are used to balance charge and support hydrogenation steps.
4. **Product evolution**: Gaseous (CO, CH_4_, C_2_H_4_) or liquid (HCOOH, CH_3_OH) products are collected.

**Principles**:

- Reaction selectivity is largely governed by the binding strength of key intermediates (e.g., *COOH, *CO).
- Cu‐based catalysts remain uniquely efficient for facilitating C─C coupling and yielding C_2+_ products.
- Competing HER must be minimized to improve CO_2_RR efficiency.

### 2.4 Analytical Techniques for CO_2_RR Evaluation

To assess catalytic performance and gain mechanistic insight, a range of characterization tools is employed:

- **Gas Chromatography (GC)**: Quantification of gaseous products like CO, CH_4_, C_2_H_4_.
- **High‐Performance Liquid Chromatography (HPLC) and NMR Spectroscopy**: Identification of liquid‐phase products such as HCOOH, CH_3_OH, and ethanol.
- **Mass Spectrometry (MS)**: Isotopic labeling (e.g., ^13^CO_2_) for tracing carbon sources and intermediates.
- **Electrochemical Techniques**:
  ○ *Linear sweep/cyclic voltammetry (LSV/CV)*: Reveal onset potential and redox processes.
  ○ *Electrochemical impedance spectroscopy (EIS)*: Provide insight into charge transport and resistance.
  ○ *Chronoamperometry/Chronopotentiometry*: Assess catalyst stability and activity over time.
- **In situ*/Operando* Spectroscopies**:
  ○ *IR, Raman, XAS, XPS*: Monitor intermediate species and active site evolution under real conditions.

## 3 Metal Nanoclusters for Photo‐ and Electrocatalytic CO_2_ Reduction

Metal NCs stand out as a powerful class of catalysts in CO_2_RR, offering distinct advantages over conventional nanoparticles (NPs). Unlike NPs, which often suffer from size variability, surface heterogeneity, and poorly defined active sites, NCs possess a well‐defined atomic structure with precisely controlled size, composition, and surface geometry. This structural precision translates to uniform active sites and reproducible, high‐fidelity catalytic performance with enhanced selectivity. One of the key strengths of NCs lies in their ability to direct the CO_2_RR pathway toward specific products by stabilizing desired reaction intermediates while suppressing competing side reactions. Their unique electronic structures, arising from their ultrasmall size and discrete energy levels, facilitate effective charge transfer and fine‐tuned interaction with CO_2_‐derived intermediates, thus improving catalytic efficiency. Moreover, their high surface area‐to‐volume ratio maximizes the exposure of active sites and enhances reactant accessibility, while minimizing mass transport limitations—a common issue in bulkier NPs. The ability of NCs to tailor intermediate binding and reaction energetics enables precise control over product distribution, making them particularly effective for targeting value‐added chemicals in CO_2_RR. When considered in unison, atomic‐level precision, enhanced charge transfer, and excellent accessibility of active sites endow NCs with remarkable catalytic proficiency. Their performance contributes meaningfully to the development of sustainable energy technologies, marking them as a pivotal platform in the pursuit of green and efficient CO_2_ conversion strategies.

### 3.1 Photocatalytic CO_2_ Reduction with Metal NCs

**Figure 4:**
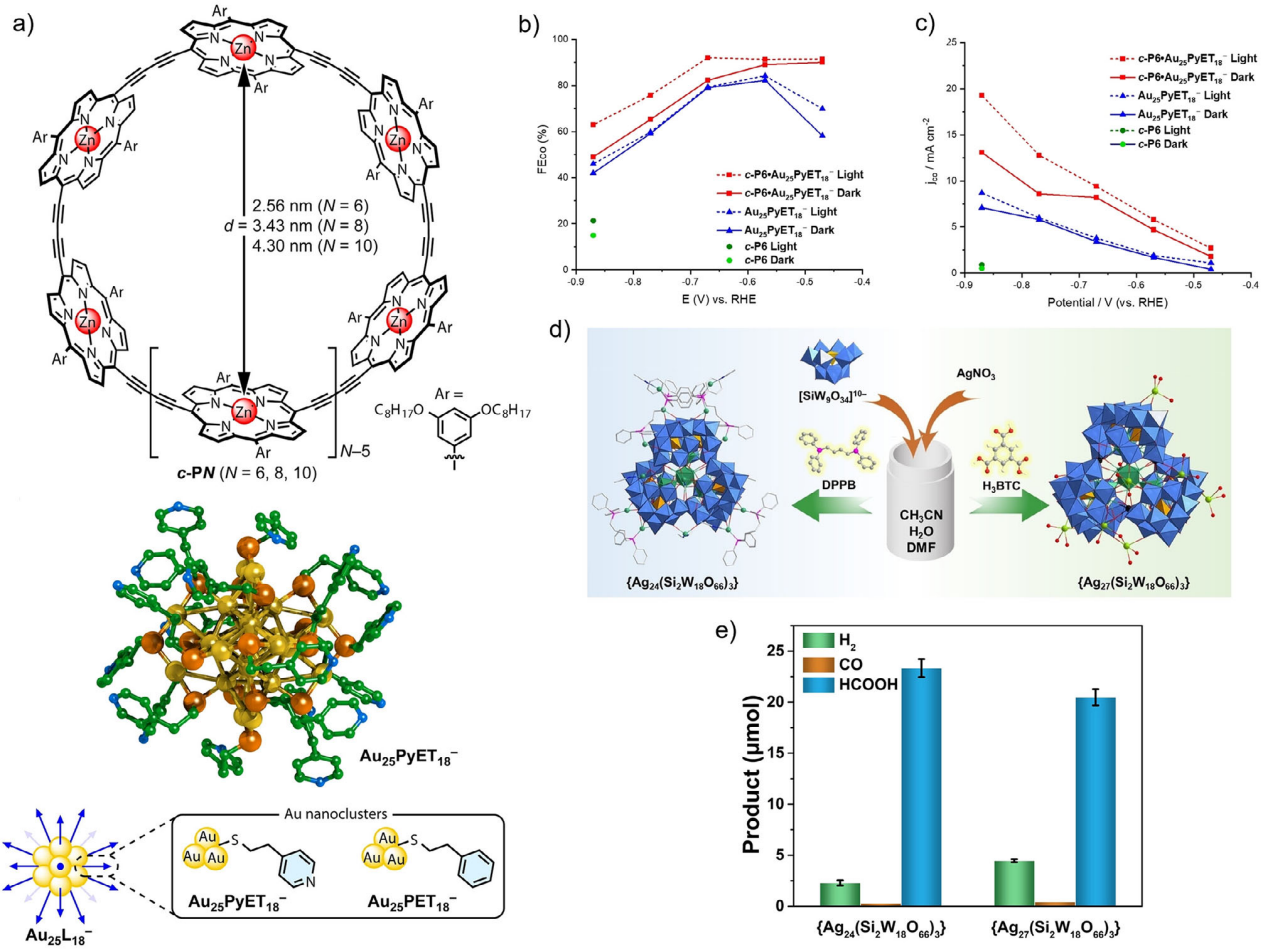
a) (top) Zinc–porphyrin nanorings linked via butadiyne bridges (*c*‐P*N*, *N* = 6, 8, 10); (bottom) anionic gold cluster Au_25_PyET_18_^−^ featuring 18 4‐pyridylethylthiolate surface ligands (PyET = 4‐pyridylethylthiolate). b) FE for CO (FE_CO_) and c) CO partial current density (*j*_CO_) of *c*‐P6, Au_25_PyET_18_^−^ and *c*‐P6·Au_25_PyET_18_^−^ under both dark and light environments. Adapted with permission from ref. [59] Copyright 2024 The Author(s). Angewandte Chemie International Edition published by Wiley‐VCH GmbH. d) Schematic representation of polyoxometalate (POM)‐stabilized silver clusters: {Ag_24_(Si_2_W_18_O_66_)_3_} and {Ag_27_(Si_2_W_18_O_66_)_3_}. e) CO_2_ photoreduction activity of {Ag_24_(Si_2_W_18_O_66_)_3_} and {Ag_27_(Si_2_W_18_O_66_)_3_}. Adapted with permission from ref. [62] Copyright 2023 Wiley‐VCH GmbH.

Metal NCs possess discrete electronic states and size‐dependent HOMO‐LUMO gaps, enabling efficient visible‐light absorption, charge separation, and multielectron transfer processes. These characteristics make them highly suitable as light harvesters or cocatalysts for photocatalytic CO_2_RR, offering both high selectivity and tunability. Among noble metal NCs, gold (Au) NCs have emerged as particularly effective photocatalytic cocatalysts due to their pronounced quantum confinement and molecular‐like energy structures. A representative example is the hydrophobic thiolate‐protected Au_25_(SR)_18_ NC cocatalyst loaded onto g‐C_3_N_4_, which exhibits a 66‐fold increase in CO formation per Au‐loading weights compared to Au NPs (∼7 nm), owing to suppressed hydrogen evolution and enhanced CO_2_ activation.^[^ ^57^ ^]^ Ligand hydrophobicity plays a decisive role: hydrophobic shells minimize water access, stabilize the COOH* intermediate, and steer the reaction toward CO_2_RR over HER. Jin et al. Moreover, hybrid NCs combining Au_25_ with Ru and Ni terpyridine complexes have displayed efficient photoinduced charge transfer, resulting in photocatalytic CO_2_‐to‐CO conversion with FEs up to 94.4% and TOFs up to in the 69989 h^−1^ range.^[^ ^58^ ^]^ In another study, a host–guest complex comprising pyridinethiol‐coated Au_25_ NC encapsulated within butadiyne‐linked zinc–porphyrin nanorings (Figure 4a) significantly enhanced both photocatalytic singlet oxygen (^1^O_2_) generation and photo‐coupled electrocatalytic CO_2_RR (Figure 4b,c).^[^ ^59^ ^]^ Tian et al. integrated Au_25_ NCs onto BiOBr nanosheets, forming a composite photocatalyst with markedly improved CO_2_ reduction performance.^[^ ^60^ ^]^ The incorporated Au NCs enhanced photogenerated charge separation and facilitated proton‐coupled electron transfer, while their anionic nature promoted CO_2_ adsorption and activation. Density functional theory (DFT) calculations confirmed that the exposed Au sites reduce the energy barriers for *COOH and *CO formation, leading to a nearly threefold increase in CO evolution rate—from 16.36 to 43.57 µmol g^−1^ h^−1^.

**Figure 5:**
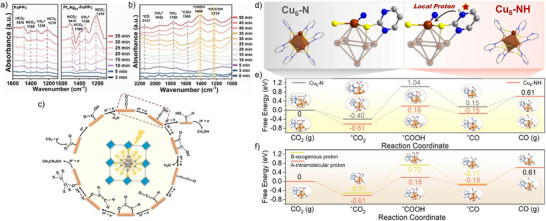
In situ FT‐IR probing of CO_2_ reduction: a) adsorption/activation sites probed under dark conditions, b) spectral fingerprints of reaction intermediates during irradiation, and c) mechanistic scheme outlining the sequence of CO_2_ photoreduction steps on Pt_1_Ag_24_–FePPc. Adapted with permission from ref. [63] Copyright 2024 Wiley‐VCH GmbH. d) Structural representations of Cu_6_ clusters and Cu coordination sites in Cu_6_─N and Cu_6_─NH. e) Computed free energy profiles for CO_2_ conversion to CO on Cu_6_─NH and Cu_6_─N, accompanied by optimized structures of the key intermediates. f) Comparison of Gibbs energy barriers for two mechanistic pathways (A: intramolecular proton transfer; B: exogenous proton supply) in CO_2_ reduction on Cu_6_–NH, with corresponding intermediate configurations. Adapted with permission from ref. [64] Copyright 2023 Wiley‐VCH GmbH.

Silver (Ag_25_) NCs, which combine discrete electronic states with surface plasmonic properties, demonstrated near‐100% selectivity toward methane in photocatalytic CO_2_RR at 100 °C.^[^ ^61^ ^]^ DFT studies indicated that CO_2_ absorption on Ag_25_ NCs is energetically favorable, while *operando* infrared spectroscopy uncovered a hydrogen‐mediated, multielectron reduction pathway proceeding via formyl and formaldehyde intermediates to surface‐bound CH*_x_* species, culminating in methane evolution. Two atomically precise polyoxometalate (POM)‐stabilized silver clusters, {Ag_24_(Si_2_W_18_O_66_)_3_} and {Ag_27_(Si_2_W_18_O_66_)_3_}, were constructed via a one‐pot solvothermal method employing distinct organic ligands (Figure 4d).^[^ ^62^ ^]^ Structural elucidation by single‐crystal X‐ray diffraction revealed unique trefoil‐propeller‐shaped {Ag_24_}^14+^ and {Ag_27_}^17+^ cores stabilized by three C‐shaped {Si_2_W_18_O_66_} units, with ∼10 delocalized valence electrons contributing to pronounced argentophilic interactions. Ultrafast transient absorption spectroscopy confirmed efficient photoinduced charge transfer between the Ag core and POM ligands, and both clusters demonstrated high selectivity for photocatalytic CO_2_ conversion to formic acid, marking the first demonstration of CO_2_RR activity in POM‐stabilized Ag clusters (Figure 4e). Huang et al. developed a hybrid catalyst by coupling single‐atom–doped Ag_24_ NCs (M = Ag, Au, Pt) with iron polyphthalocyanine through electrostatic assembly, enabling fine modulation of the electronic structure and charge separation properties.^[^ ^63^ ^]^ Among the variants, Pt_1_Ag_24_–FePPc displayed the strongest asymmetric charge distribution, which facilitated CO_2_ adsorption and activation and delivered the highest methanol and ethanol yields under photocatalytic conditions. Mechanistic understanding was gained from in situ FT‐IR measurements showing CO_2_ activation in the dark (Figure 5a), time‐resolved spectral changes under illumination (Figure 5b), and the proposed intermediate‐driven reaction pathway (Figure 5c).

Atomically precise copper (Cu) clusters are compelling candidates for photocatalytic CO_2_RR owing to their low cost, adjustable electronic configurations, and well‐defined active sites, making them excellent molecular models for elucidating structure–function correlations. In a recent study, Dong et al. developed a crystalline Cu─S─N cluster catalyst (Cu_6_─NH) incorporating protonated pyrimidine N─H groups that function as localized proton donors (Figure 5d).^[^ ^64^ ^]^ The catalyst displayed strong photoredox activity, excellent structural integrity, visible‐light absorption, and nearly 100% selectivity toward CO generation. Combined experimental and theoretical investigations—including isostructural analog studies, in situ diffuse reflectance infrared Fourier‐transform spectroscopy (DRIFTS), and DFT calculations (Figure 5e,f)—demonstrated that the N─H moieties serve as proton relays, significantly reducing the energy barrier for *COOH formation, the rate‐limiting step, thereby improving photocatalytic efficiency.

Polyoxo‐titanium clusters (PTCs) are enticing candidates for photocatalytic CO_2_ reduction due to their tunable molecular structures, well‐defined active sites, and TiO_2_‐like electronic properties that enable visible‐light absorption, efficient charge separation, and structural elucidation at the atomic level. Liu et al. developed three PTC‐based photocatalysts—Ti_6_–PPOA, Ti_8_–Fcdc, and Ti_6_–Fcdc—by functionalizing the Ti‐oxo cores with phenylphosphonic acid (PPOA) or 1,1′‐ferrocenedicarboxylic acid (Fcdc).^[^ ^65^ ^]^ The incorporation of Fcdc, a redox‐active ligand, significantly extended light absorption into the visible range and promoted strong ligand‐to‐metal charge transfer, enhancing photocatalytic performance. Under visible‐light irradiation and using triisopropanolamine (TIPA) as a sacrificial donor in water, Ti_6_–Fcdc achieved a remarkable CO_2_‐to‐formate selectivity of 97.5% and an activity of 350.00 µmol g^−1^ h^−1^—the highest reported among PTC‐based CO_2_RR systems.

Heterometallic clusters are highly attractive for photocatalytic CO_2_RR due to their tunable electronic structures, synergistic metal–metal interactions, and atomically precise active sites that enable controlled redox behavior and multielectron transfer processes. Guo et al. reported a groundbreaking series of all‐inorganic core–shell cobalt polyoxoniobates (Co–PONbs), including {Co_12_Nb_38_O_132_}, {Co_20_Nb_34_O_128_}, {Co_26_Nb_36_O_140_}, and {Co_33_Nb_54_O_188_}, representing the largest Co–PONbs and the most cobalt‐rich polyoxometalates identified to date.^[^ ^66^ ^]^ These molecular clusters feature well‐defined core–shell architectures with cobalt oxide cores encapsulated by niobate shells, closely resembling structural motifs of spinel‐type Co_3_O_4_. The mixed‐valence Co^2+^/Co^3+^ cores act as discrete molecular analogues of Co_3_O_4_, and the resulting bimetallic clusters exhibit promising activity and stability for visible‐light‐driven CO_2_RR, achieving a CO/H_2_ generation rate of 33.42/25.52 µmol h^−1^ and a TON of 491.17 within 1 h of illumination.

### 3.2 Electrocatalytic CO_2_ Reduction with Metal NCs

The gold nanocluster [Au_25_(PET)_18_]^−^ has emerged as a cornerstone in electrocatalytic CO_2_RR studies. Kauffman and colleagues demonstrated that Au_25_ exhibits an exceptionally low CO_2_RR onset overpotential (∼90 mV versus RHE), which is ∼200–300 mV lower than that observed for larger Au NPs and bulk Au.^[^ ^67^ ^]^ At –1.0 V versus RHE, the cluster achieved near‐quantitative FE along with a peak CO generation rate of 1.26 mmol cm^−2^ h^−1^—outperforming larger NPs by 7–700×. This enhanced activity is attributed to its anionic nature, which facilitates CO_2_ adsorption, and its atomically precise surface environment that promotes C═O bond activation and H_ads_ formation. Crucially, the anionic form of Au_25_^−^ promotes CO_2_ reduction activity relative to neutral or cationic variants by stabilizing co‐adsorbed CO_2_ and H^+^ reactants.^[^ ^68^ ^]^

**Figure 6:**
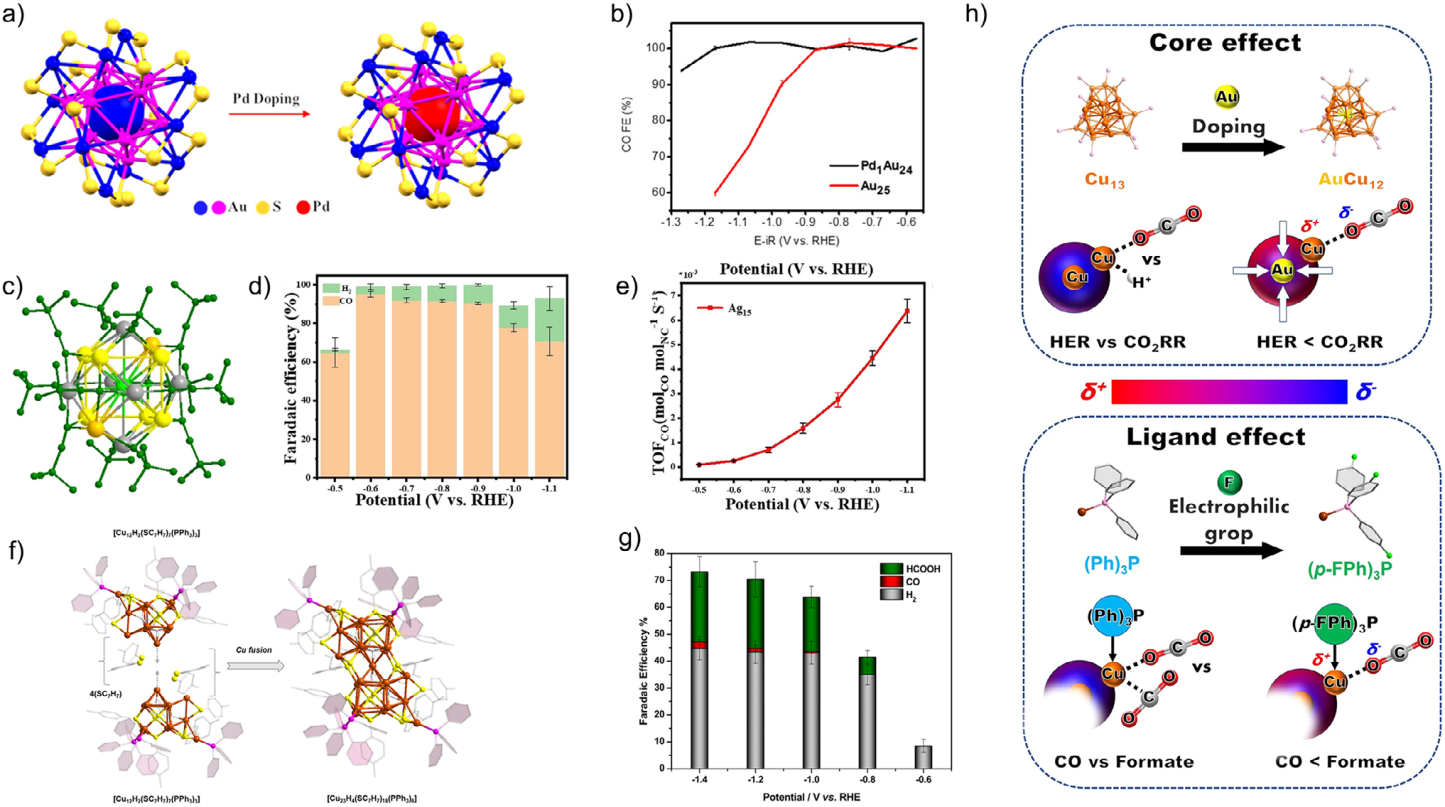
a) Structural configurations of Au_25_ and Pd_1_Au_24_ NCs (R substituents excluded). b) FEs for CO evolution over the respective catalysts. Adapted with permission from ref. [69] Copyright 2023 The Authors. c) Structural depiction of Ag_15_(C≡C‐^t^Bu)_12_^+^ NC. d) FE_CO_ at different potentials in 0.5 M KHCO_3_. e) TOF for CO_2_RR. Adapted with permission from ref. [79] Copyright 2021 Wiley‐VCH GmbH. f) Structure of Cu_23_ NC, assembled by fusion of two identical subunits through a central Cu atom. For clarity, hydrogen atoms are omitted. Atom colors: Cu (brown), S (yellow), P (violet), H (white), and C (gray); PPh_3_ phenyl rings are shaded for emphasis. g) FE profiles for CO_2_RR over Cu_23_/CB at different applied potentials, with error bars indicating mean ± SD (*n* = 3). Adapted with permission from ref. [91] Copyright 2025 The Authors. Published by American Chemical Society. h) Core (Cu_13_ versus AuCu_12_) and ligand ((Ph)_3_P versus (*p*‐FPh)_3_) effects comparison. Adapted with permission from ref. [84] Copyright 2022, The Author(s).

Single‐atom and heterometal doping of Au NCs has emerged as a powerful strategy to tune electronic structure, modulate adsorption energetics, and enhance selectivity in electrocatalytic CO_2_RR. Li et al. demonstrated that central Pd atom substitution in Au_25_(SC_2_H_4_Ph)_18_ (yielding Pd_1_Au_24_) preserves the cluster framework while drastically suppressing the competing hydrogen evolution reaction (HER) (Figure 6a).^[^ ^69^ ^]^ The Pd‐doped NC achieves nearly 100% FE for CO over a broad potential range (–0.6 to –1.2 V versus RHE), in contrast to the rapid performance decline observed for undoped Au_25_ beyond –0.9 V (Figure 6b). DFT analysis attributes this enhancement to the stabilization of thiolate‐bound sulfur active sites under cathodic conditions, along with suppression of HER‐prone undercoordinated Au sites. In another approach, Chen et al. demonstrated that surface modification of Au_60_ via site‐selective introduction of a bridging μ_4_‐sulfur atom transforms outer kernel Au atoms into catalytically favorable staple motifs without disrupting the cluster core.^[^ ^70^ ^]^ The modified Au_60_S_6_ achieved >95% CO selectivity across all tested potentials, which DFT analysis linked to an upshifted d‐band center that facilitates intermediate binding and hinders CO desorption. Deng et al. synthesized a well‐defined AuCu nanoalloy cluster, [Au_15_Cu_4_(DPPM)_6_Cl_4_(C≡CR)1]^2+^ (Au_15_Cu_4_), comprising two interpenetrating icosahedral subunits.^[^ ^71^ ^]^ The Cu‐doped cluster exhibits over 90% FE_CO_ and an industrially relevant CO partial current density of –413 mA cm^−2^ in a membrane electrode assembly (MEA) cell—more than double that of its Au‐only analogue, Au_18_. DFT analysis attributes this performance to synergistic Au–Cu dual sites that modulate the d‐band center and enhance intermediate binding under high current densities. Complementing these efforts, Li and co‐workers achieved atomic‐level tuning of Au_23_ by replacing two surface Au atoms with Cd, forming Au_19_Cd_2_.^[^ ^72^ ^]^ This Cd‐doped NC exhibited doubled CO_2_RR selectivity (90–95%) over its undoped counterpart and delivered a record‐high activity of 2200 mA mg^−1^ at –1.0 V versus RHE. DFT studies revealed that partial ligand removal exposes catalytically active S sites and significantly lowers the thermodynamic barrier for CO formation (by 0.74 eV), underscoring the unique Au–Cd synergism in promoting CO_2_ activation. Despite their outstanding activity, Au‐based NCs suffer from low atom‐utilization efficiency as many Au atoms are catalytically inactive. To address this, Seong et al. transplanted isolated Au active sites into a non‐precious Ni_4_ NC host, switching selectivity from H_2_ (Ni_4_) to CO (Au_4_–Ni_2_).^[^ ^73^ ^]^ This design achieved an exceptional TOF (206 mol_CO_/mol_NC_/s) and mass activity (25228 A/g_Au_) at an overpotential of just 0.32 V, with stable CO_2_RR performance over 25 h. A study by Su et al. represents a rare example where Cu and Ag were doped into gold nanoclusters at the same atomic site, allowing a direct comparison of their intrinsic effects without structural bias.^[^ ^74^ ^]^ By preparing atomically precise Au_8_Cu_1_ and Au_8_Ag_1_ clusters, they showed that subtle differences between dopants can dramatically alter CO_2_RR activity, with Cu promoting far more efficient CO formation than Ag. This work provides a powerful model system for disentangling atomic‐level synergies in alloy nanoclusters and guiding rational catalyst design.

The structural configuration and ligand chemistry of Au NCs critically influence their electrocatalytic CO_2_RR performance. Li et al. systematically investigated ligand effects by comparing Au_25_ NCs bearing identical metallic kernels but distinct anchoring atoms (S or Se) and carbon tails.^[^ ^75^ ^]^ While the alkyl tail had negligible influence, the anchoring atom played a decisive role: sulfur ligands enhanced CO selectivity by stabilizing key intermediates, lowering the energy barriers for *COOH and *CO formation by 0.26 and 0.43 eV, respectively, compared to selenium. In parallel, size effects were explored by Seong et al.^[^ ^76^ ^]^ and Li et al.,^[77^ ^]^ who showed that increasing the size of Au NCs (Au_25_ → Au_38_ → Au_144_) increases active site density and CO partial current, yet the CO Faradaic efficiency remains size‐independent—highlighting that surface‐to‐volume ratio governs activity, while selectivity is controlled by surface electronic configuration. Furthermore, comparison of two structurally distinct Au_38_ isomers (Au_38_Q and Au_38_T) revealed that even subtle geometric rearrangements can profoundly alter CO_2_RR outcomes. Au_38_Q exhibited superior CO selectivity and activity, attributed to more favorable *COOH formation energetics as revealed by DFT. Collectively, these studies underscore that beyond composition and size, the identity of surface ligands and atomic‐level geometry critically dictate CO_2_ activation pathways, intermediate stabilization, and overall electrocatalytic efficiency.

Ag NCs, like their gold counterparts, offer atomically precise structures and tunable surface chemistry, making them promising candidates for electrocatalytic CO_2_RR. Their relatively lower cost and distinct d‐band characteristics open alternative avenues for catalyst design. Seong and co‐workers compared Ag_25_(SR)_18_ with Au_25_(SR)_18_ and found Ag_25_ to exhibit substantially lower CO_2_RR activity due to its higher limiting potential.^[^ ^78^ ^]^ However, by constructing a core–shell AuAg_12_@Au_12_(SR)_18_ NC, they achieved dramatically enhanced CO selectivity and stability at industrially relevant current densities (200 mA cm^−2^) in a zero‐gap electrolyzer. Qin et al. introduced a homoleptic alkynyl‐protected Ag_15_ NC (Ag@Ag_8_@Ag_6_ BCC core) (Figure 6c), which self‐assembles during crystallization and delivers ∼95% CO Faradaic efficiency at −0.6 V (Figure 6d), with a TOF of 6.37 s^−1^ (Figure 6e) and strong operational stability.^[^ ^79^ ^]^ DFT calculations identified undercoordinated Ag atoms, revealed upon partial ligand stripping, as the active sites. Extending this design, a homoleptic alkynyl‐protected Ag_32_ NC with a C_3_‐symmetric Ag_17_ core exhibited outstanding CO selectivity (∼96.4% at –0.8 V) and dual catalytic functionality for 4‐nitrophenol reduction.^[^ ^80^ ^]^ The low energy barrier for *COOH formation and enhanced ligand effects were attributed to the alkynyl shell. Yoo and co‐workers further investigated core‐atom effects in structurally identical PtAg_24_ and AuAg_24_ NCs, synthesized via ligand‐induced restructuring and galvanic exchange.^[^ ^81^ ^]^ While PtAg_24_ displayed poor CO selectivity (∼30%), the Au‐centered cluster achieved 90% CO selectivity with partial current densities up to −202.2 mA cm^−2^ in MEA cells, maintaining performance over 24 h. Operando vibrational spectroscopy and DFT revealed that the central Au atom tunes *CO intermediate binding, highlighting the core metal as a decisive factor in eCO_2_RR selectivity and activity.

Cu NCs have emerged as powerful model systems for uncovering structure–function relationships in electrocatalytic CO_2_RR, owing to their earth‐abundance, tunable redox chemistry, and unique ability to generate a wide range of products—from two‐electron species (H_2_, CO, HCOOH) to multielectron hydrocarbons (CH_4_, C_2_H_4_, C_2+_). Unlike Au and Ag NCs, which primarily yield CO, Cu NCs demonstrate rich catalytic diversity dictated by atomic‐level structure, ligand coordination, and oxidation state. Hydride incorporation is particularly impactful. Tang et al. demonstrated that ligand‐protected Cu_32_H_20_ clusters drive HCOOH formation via a lattice‐hydride mechanism at low overpotential, as supported by DFT calculations.^[^ ^82^ ^]^ Zang et al. further revealed that structural isomerism in Cu_8_ clusters—specifically, ditetrahedral versus cubic geometries—substantially influences *HCOO intermediate stabilization, with the ditetrahedral cluster achieving ca. 92% FE for formate.^[^ ^83^ ^]^ Doping and ligand engineering offer another route for tuning activity and selectivity. Wang et al. employed [M@Cu_24_H_22_(PR_3_)_12_]^+^ clusters (M = Cu or Au; R = PPh_3_ or p‐FPPh_3_) to show that Au doping promotes CO_2_RR while suppressing HER, with fluoro‐substituted phosphines enhancing CO_2_‐to‐formate selectivity (Figure 6h).^[^ ^84^ ^]^ Ma et al. revealed that precisely engineered Cu_3_H_3_ units within the AuCu_24_ NC serve as key active motifs that drive CO_2_ reduction toward C_2+_ products by stabilizing critical C─C coupling intermediates.^[^ ^85^ ^]^ Combined spectroscopic, isotopic, and theoretical analyses highlighted the essential role of lattice hydrides in tuning selectivity, offering a new molecular‐level strategy for designing copper‐based catalysts for multicarbon conversion. Shen et al. reported a [Cu_26_]^+^ cluster featuring quintuple ligands—including phosphines, carboxylates, alkynes, hydrides, and alcohols—that exhibited high CO selectivity, attributed to its intricate hybrid surface.^[^ ^86^ ^]^ Recent studies also target hydrocarbon generation. Huang et al. introduced Cu_6_(MBD)_6_ clusters (MBD = 2‐mercaptobenzimidazole), where S/N bidentate coordination induces Cu–S_2_N_1_ active sites with modulated 3d orbital occupancy, promoting selective CH_4_ and C_2_H_4_ production (65.5% FE at −1.4 V versus RHE).^[^ ^87^ ^]^ In a related effort, Li et al. designed Cu_4_ and Cu_8_ clusters with MMI (2‐mercapto‐1‐methylimidazole) ligands that maintain Cu^+^ centers during catalysis, enabling CH_4_ and C_2_^+^ generation with excellent stability.^[^ ^88^ ^]^ Ligand control also affects product distributions and catalyst longevity. Negishi et al. demonstrated that small changes in thiolate ligands or geometric motifs in Cu_11_, Cu_14_, and Cu_18_ clusters result in drastic shifts in selectivity between H_2_, HCOOH, and hydrocarbons.^[^ ^89^, ^90^ ^]^ In a landmark study, they achieved Cu(0)‐based [Cu_23_H_4_(SR)_18_(PPh_3_)_6_] clusters stabilized by Cu(I)‐based cages (Figure 6f), yielding high structural stability and selective HCOOH production (Figure 6g).^[^ ^91^ ^]^ Most recently, Zhu et al. synthesized isostructural Cu_13_ and Cu_14_ NCs differing by a single Cu atom.^[^ ^92^ ^]^ This minor perturbation significantly altered the electronic structure, enabling Cu_14_ to selectively generate CH_4_ and C_2_H_4_ (54.3% FE), while Cu_13_ remained inactive. Collectively, these studies establish Cu NCs as a versatile class of atomically precise electrocatalysts for CO_2_RR, where subtle variations in structure, oxidation state, ligand shell, and atomic composition enable precise control over reaction pathways and product selectivity. Table 1 summarizes the critical structural parameters of NCs and their mechanistic roles in dictating CO_2_ reduction activity and selectivity.

**Table 1:** Summary of key structural factors in NCs that influence photo‐ and electrocatalytic CO_2_ reduction. Structural variations—including cluster size, composition, ligands, doping, lattice strain, and confinement—modulate electronic structure (e.g., d‐band center, charge density), charge separation efficiency, intermediate stabilization, and metal–metal synergistic effects, thereby dictating activity and selectivity toward C_1_ or C_2+_ products.

Structural features of nanoclusters	Key factor influenced	Mechanistic effect on CO_2_RR	Representative references
Cluster size (number of atoms)	Quantum confinement, electronic shell closure	Alters HOMO–LUMO gap, redox potential, and product selectivity (e.g., smaller NCs favor C_1_, larger NCs facilitate C─C coupling)	[67, 68, 69, 71, 76, 77, 79]
Core composition (monometallic vs bimetallic/alloy)	d‐band center, synergistic metal effects	Tuning the d‐band modifies CO adsorption strength and *CO dimerization; synergistic interactions enhance selectivity toward C_2+_ products	[72, 73, 74, 79, 83, 84, 88]
Surface ligands (type, electronic and steric effects)	Surface charge distribution, hydrophobicity, intermediate stabilization	Ligands modulate local electrostatic fields and hydrophobic/hydrophilic balance, altering *COOH and *CO binding and suppressing competing HER	[63, 65, 70, 80, 81, 84, 87, 89, 90, 91]
Heteroatom doping (e.g., Cu, Ag, Pt substitution)	Charge density redistribution, electronic localization	Substitution induces asymmetric charge distribution, tailoring intermediate stabilization (e.g., Cu favors *CO binding vs Ag)	[74, 77, 83, 84, 88]
Cluster–support or cluster–framework coupling	Charge separation efficiency, interfacial electron transfer	Enhanced electron‐hole separation and directional transfer through strong coupling with MOFs/COFs or conductive supports	[60, 62, 65, 66, 87]
Lattice strain/hydride incorporation	Electronic structure modulation, stabilization of intermediates	Strained lattices or lattice hydrides stabilize *CO and CO─COH, promoting C─C coupling and C_2+_ products	[82, 85]
Pore confinement/microenvironment engineering	Mass transport, intermediate diffusion, local proton concentration	Confinement in frameworks concentrates CO_2_ near active sites, modulates PCET steps, and biases toward desired products	[61, 62, 64, 66, 80]
Crystallinity and surface atom coordination	Density of active sites, adsorption geometry	Under‐coordinated atoms or specific exposed facets provide sites for stronger *CO binding and facilitate multielectron transfer	[69, 70, 73, 76, 84, 87]

## 4 Extended Frameworks for Photo‐ and Electrocatalytic CO_2_ Reduction: Current State‐of‐the‐Art

Extended porous reticular frameworks, exemplified by MOFs and COFs, offer several key advantages over discrete NCs in photo‐ and electrocatalytic CO_2_ reduction. Unlike isolated NCs, these crystalline porous architectures provide long‐range structural order, permanent porosity, and modularity that allow precise spatial arrangement of catalytic sites and functional motifs within well‐defined, accessible environments. Their highly tunable frameworks enable the incorporation of photoactive and redox‐active units, enhanced mass transport, and favorable charge separation pathways, all of which contribute to superior catalytic efficiency and product selectivity. Moreover, their extended conjugation and structural robustness under electrochemical or photochemical conditions often translate to improved long‐term durability. In the following section, we highlight recent cutting‐edge developments in the design and application of such reticular frameworks for CO_2_ conversion under light and electrochemical stimuli.

### 4.1 Light‐Harvesting Frameworks: Photocatalytic CO_2_ Reduction Using MOFs and COFs

*MOFs*: Zirconium‐ and transition‐metal‐based MOFs have emerged as a versatile platform for photocatalytic CO_2_RR, owing to their tunable pore environments, high surface areas, and structural stability. The incorporation of active metal sites, controlled defect engineering, heterojunction formation, and tailored light‐absorbing ligands have significantly broadened their applicability across product pathways—from CO to formic acid, methane, and alcohols.

**Figure 7:**
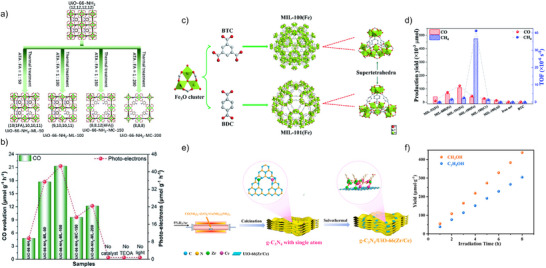
a) Structural diagrams of defect‐free and defective UiO‐66‐NH_2_ frameworks, including linker and cluster vacancies. b) Photocatalytic CO_2_‐to‐CO conversion and photoinduced charge carrier dynamics across different defect types. Adapted with permission from ref. [93] Copyright The Royal Society of Chemistry 2022. c) Structural illustration of MIL‐100(Fe) and MIL‐101(Fe), showing Fe_3_O nodes linked by BTC or BDC ligands. Atom colors: O = red, C = gray, Fe = green. d) CO_2_ photoreduction to CO and CH_4_ and associated TOF metrics. Adapted with permission from ref. [101] Copyright The Royal Society of Chemistry 2020. e) Preparation scheme of g‐C_3_N_4_/UiO‐66(Zr/Ce). f) CH_3_OH and C_2_H_5_OH output from photocatalytic CO_2_ conversion. Adapted with permission from ref. [107] Copyright 2023 American Chemical Society.

CO generation from CO_2_ over MOF‐based photocatalysts has been significantly improved through precise engineering of structural defects, metal–ligand interactions, solubility‐based enhancements, and many other strategies. Wang et al. demonstrated that linker and cluster defects in UiO‐66‐NH_2_ (Figure 7a) systematically modulate the light absorption energy (*E*_abs_) and ligand‐to‐metal charge transfer energy (*E*_LMCT_), leading to a 2.2‐fold increase in CO yield (Figure 7b) with missing‐linker (ML) defects compared to missing‐cluster (MC) variants.^[^ ^93^ ^]^ Li et al. developed a Pt‐loaded Ni‐MOF that achieved 9.57% CO_2_ conversion efficiency at 940 nm by synergistically combining open Ni sites for CO_2_ activation and Pt sites for H_2_ dissociation. The dissociated H_2_ spills over to Ni, enabling thermal reduction of CO_2_ to CO and CH_4_.^[^ ^94^ ^]^ Additionally, Fang et al. introduced a soluble UiO‐66 as a co‐catalyst to boost CO_2_‐to‐CO photocatalysis, yielding 30.6 µmol h^−1^ using only 5.0 mg of sample—approximately 1.5 times higher than the insoluble analogue.^[^ ^95^ ^]^ This enhancement is attributed to the preserved framework in the soluble UiO‐66, which improves CO_2_ chemisorption and facilitates more efficient CO_2_ activation and reduction.

Hao et al. achieved high selectivity for formic acid by introducing Ir single atoms into porous MOF membranes, attaining near‐perfect selectivity and an apparent quantum efficiency of 15.76% under humid CO_2_ gas.^[^ ^96^ ^]^ Guo et al. improved charge transfer by modulating the MOF's photoexcited behavior from triplet metal‐to‐ligand charge transfer (^3^MLCT) to triplet intraligand (^3^IL) transitions, achieving a benchmark HCOOH output of 4807.6 µmol g^−1^.^[^ ^97^ ^]^ Hierarchical MOF@MOF architectures by Huang et al. further extended visible‐light response and improved charge separation, achieving HCOOH production of 146.0 µmol g^−1^ h^−1^ without sacrificial agents.^[^ ^98^ ^]^ Atomically dispersed Fe sites in 2D Fe/Ti‐BPDC MOFs also showed high selectivity (>99.7%) and yield (703.9 µmol g^−1^ h^−1^), facilitated by rapid electron transfer and CO_2_ activation.^[^ ^99^ ^]^

CH_4_ generation from CO_2_ demands multielectron processes and strong protonation control. Li et al. constructed a core–shell Cu_3_(BTC)_2_@TiO_2_ system, where the MOF core served as a gas trap while the semiconductor shell promoted exciton generation and charge transfer, boosting CH_4_ selectivity.^[^ ^100^ ^]^ MIL‐100(Fe) outperformed MIL‐101(Fe) in CH_4_ generation under visible light, ascribed to ligand‐dependent cluster environment tuning (Figure 7c,d).^[^ ^101^ ^]^ Huang et al. revealed that Fe‐POMOFs produce CH_4_ selectively in photocatalysis and CO in electrocatalysis by manipulating electron flow between the porphyrin core and the POM cluster.^[^ ^102^ ^]^ The SYD‐1‐CuNi system, integrating Ru‐based metalloligands with postsynthetic metalation, enabled simultaneous CO_2_‐to‐CH_4_ conversion, HER, and benzyl alcohol‐to‐benzaldehyde oxidation.^[^ ^103^ ^]^ In a recent contribution, Huang et al. further enhanced CH_4_ selectivity via Au nanorods confined in porphyrin MOFs, exploiting plasmonic effects to amplify *CO and *CHO intermediates and clarify protonation steps via kinetic isotope effects.^[^ ^104^ ^]^

Alcohol formation, requiring efficient *CHO and C─C coupling, was enabled by Cu single atoms anchored on UiO‐66‐NH_2_, as shown by Wang et al., achieving CH_3_OH and C_2_H_5_OH yields of 5.33 and 4.22 µmol h^−1^ g^−1^, respectively.^[^ ^105^ ^]^ Li et al. developed a 2D NiZrCu‐BDC nanosheet, yielding 41.05 µmol h^−1^ g^−1^ CH_3_OH and 36.62 µmol h^−1^ g^−1^ C_2_H_5_OH, facilitated by surface charge enrichment and efficient electron delivery.^[^ ^106^ ^]^ Their follow‐up work with g‐C_3_N_4_/UiO‐66(Zr/Ce) hybrids (Figure 7e) established strong interfacial electron transport via N─Zr/Ce─O bonds, achieving CH_3_OH and C_2_H_5_OH production at 54.71 and 38.10 µmol h^−1^ g^−1^, respectively (Figure 7f), without sacrificial agents.^[^ ^107^ ^]^ The introduction of Ce doping further concentrated electrons around Ce sites, favoring multielectron transfer steps crucial for alcohol synthesis. These advances underscore the importance of precise structural modulation—ranging from defect engineering and single‐atom incorporation to hybrid heterojunctions and photophysical reconfiguration—in elevating the selectivity and efficiency of MOF‐based photocatalysts. Continued development of MOFs with tailored optoelectronic properties and synergistic active sites is expected to unlock new horizons in selective solar‐driven CO_2_ conversion.

*COFs*: COFs are particularly well‐suited as photocatalysts for CO_2_RR due to their highly tunable structures and multifunctional properties. Built from organic building blocks linked by strong covalent bonds into extended crystalline frameworks, COFs exhibit exceptional porosity and long‐range π‐conjugation, which facilitate efficient light harvesting and charge transport—both critical for photocatalytic processes. Their modularity allows precise incorporation of light‐absorbing units such as porphyrins, phthalocyanines, or donor–acceptor motifs, enabling strong absorption in the visible region and efficient generation of photoexcited charge carriers. Furthermore, the ordered and tunable pore architecture enhances CO_2_ uptake and diffusion, providing abundant and accessible active sites for catalytic conversion. The chemical environment around these sites can be rationally designed to stabilize key intermediates and lower activation barriers, thereby improving selectivity and efficiency. Additionally, the robust and chemically stable nature of COFs ensures sustained photocatalytic activity under reaction conditions. Collectively, these features—porosity, crystallinity, conjugation, and structural tunability—make COFs a compelling platform for designing next‐generation photocatalysts for efficient and selective CO_2_ reduction.

Photocatalytic CO_2_ reduction using COFs was pioneered with N_3_‐COF, an azine‐linked framework previously developed by Vyas et al.^[^ ^108^ ^]^ Fu and co‐workers demonstrated an efficient, metal‐free strategy for reducing CO_2_ with water under visible light irradiation, using N_3_‐COF as the catalyst to methanol under irradiation of visible light without the use of sacrificial agents.^[^ ^109^ ^]^ Remarkably, this system achieved a methanol yield of 13.7 µmol g^−1^ over 24 h. Even at a low CO_2_ concentration of 1%, the catalyst maintained high performance, producing 9.9 µmol g^−1^ of methanol. The N_3_‐COF retained its crystallinity and catalytic activity over five consecutive cycles, highlighting its robustness. Under comparable reaction conditions, azine‐linked COFs exhibited superior photocatalytic activity for CO_2_ reduction compared to g‐C_3_N_4_, positioning this study as a landmark in metal‐free, sustainable photocatalysis and highlighting COFs as highly promising organic semiconductors for solar fuel production. In a recent study, Ning et al.^[^ ^110^ ^]^ introduced a hybrid photocatalyst combining N_3_‐COF with MoS_2_, achieving enhanced CO_2_ reduction under aerobic conditions. Notably, at an O_2_ concentration of 20%, akin to atmospheric levels, the system achieved a CO production rate of 28 µmol g^−1^ h^−1^, surpassing the performance observed under pure CO_2_ conditions.

**Figure 8:**
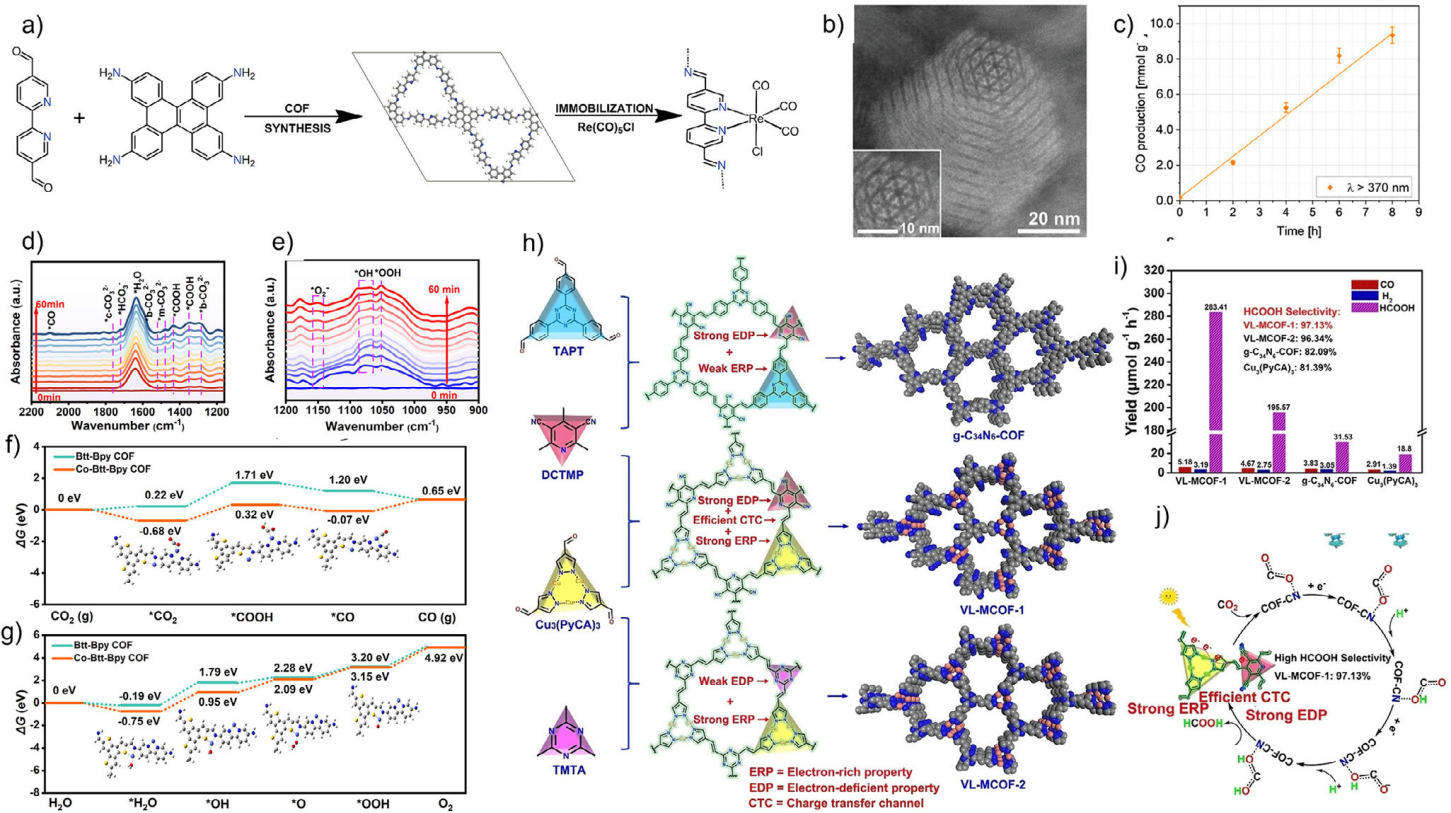
a) Construction of bpyDBC COF followed by incorporation of Re^I^(CO)_5_Cl to form Re^I^bpyDBC COF. b) Visualization of the COF‐integrated Re^I^ motifs using HAADF‐STEM imaging. c) Photocatalytic CO generation by Re^I^bpyBC COF upon *λ* > 370 nm illumination. Adapted with permission from ref. [113] Copyright 2025 The Author(s). Small published by Wiley‐VCH GmbH. In situ ATR FT‐IR measurements of d) CO_2_ and H_2_O, and e) Ar and H_2_O interactions with Co‐Btt‐Bpy COF under subsequent light irradiation. Energy diagrams from DFT calculations illustrating the catalytic pathways for f) CO_2_ photoreduction and g) water oxidation on functionalized versus pristine Btt‐Bpy COFs. Adapted with permission from ref. [115] Copyright 2025 American Chemical Society. h) Illustration of the Knoevenagel condensation‐based synthesis and resulting structures of g‐C_34_N_6_‐COF, VL‐MCOF‐1, and VL‐MCOF‐2. i) Photoreduction product analysis showing HCOOH selectivity and yield profiles for all tested COFs and Cu_3_(PyCA)_3_. j) Mechanistic depiction of the photocatalytic CO_2_‐to‐HCOOH process facilitated by VL‐MCOF‐1. Adapted with permission from ref. [118] Copyright 2023 Wiley‐VCH GmbH.

Recognized for their exceptional photoelectric characteristics, 2,2′‐bipydine‐based metal complexes are potent candidates for CO_2_ reduction. Yang et al. incorporated the tricarbonylchloro(bipyridyl) Re complex Re(bpy)(CO)_3_Cl moiety to a triazine‐based COF through post‐synthetic modification.^[^ ^111^ ^]^ The resulting Re‐COF retained crystallinity and demonstrated a 22‐fold improvement in CO production (15 mmol g^−1^ over >20 h) under visible light (*λ* ≥ 420 nm) with TEOA as sacrificial donor, compared to its homogeneous counterpart, while maintaining 98% selectivity and good reusability. In another study, Fu and colleagues^[^ ^112^ ^]^ constructed a sp^2^‐carbon‐linked COF with tethered Re(bpy)(CO)_3_Cl complexes that delivered robust photocatalytic CO_2_ reduction performance in the presence of TEOA as sacrificial donor under visible‐light exposure (*λ* ≥ 420 nm). The as‐synthesized catalyst, Re‐Bpy‐sp^2^c‐COF, attained a notable CO output of 1040 µmol g^−1^ h^−1^ and 81% selectivity over H_2_ for a period of 17.5 h illumination. Leveraging the extended π‐conjugation and planar geometry of the dibenzochrysene unit, Spies et al. designed a stable, crystalline Re^I^‐functionalized COF (Re^I^bpyDBC COF) for photocatalytic CO_2_ reduction (Figure 8a).^[^ ^113^ ^]^ Precise incorporation of Re(bpy)(CO)_3_Cl sites was achieved via predefined ligation points, and HAADF‐STEM confirmed the ordered arrangement of Re centers (Figure 8b). The hybrid material exhibited efficient CO evolution of 1.16 mmol g^−1^ h^−1^ under UV–vis light with BIH as sacrificial donor (Figure 8c) and maintained moderate activity under visible light for over 72 h, comparable to the most durable COF photocatalysts. In another work, Zhong et al. introduced a Ni‐TpBpy COF, where Ni centers are incorporated into the TpBpy framework, serving as active sites for photocatalytic CO_2_ reduction to CO.^[^ ^114^ ^]^ Despite exhibiting a lower BET surface area compared to the pristine TpBpy COF, Ni‐TpBpy demonstrated a slightly enhanced CO_2_ adsorption capacity, indicating a stronger affinity for CO_2_ molecules. Utilizing [Ru(bpy)_3_]Cl_2_ as a photosensitizer and TEOA as an electron donor in aqueous solution under visible light, the Ni‐TpBpy catalyst achieved a CO production of 4057 µmol g^−1^ within 5 h, with a high selectivity of 96% over H_2_. Ni‐TpBpy reached a turnover number of 13.62 for CO evolution and an AQE of 0.3% at 420 nm. Notably, at a CO_2_ concentration of 10%, it still produced 915 µmol g^−1^ of CO with 76% selectivity over 4 h. The study's findings, corroborated by control experiments and DFT calculations, underscore the importance of nickel integration in the TpBpy framework for achieving high catalytic activity and selectivity for CO_2_ reduction.

Developing dual‐functional photocatalysts capable of concurrently driving CO_2_ reduction and water oxidation remains a substantial obstacle. An ideal system should promote CO_2_ and H_2_O adsorption, contain distinct sites for both reduction and oxidation reactions, and facilitate efficient electron–hole migration. In a recent advancement, Xu et al. reported a donor–acceptor (D–A) COF strategically functionalized with Co or Ni ions to introduce N‐metal–N and N‐metal–S coordination environments that functioned as dual catalytic centers for reduction and oxidation, respectively.^[^ ^115^ ^]^ The resulting Co‐Btt‐Bpy COF achieved an impressive CO evolution rate of 9,800 µmol g^−1^ h^−1^ and O_2_ evolution of 242 µmol g^−1^ h^−1^ under visible light, outperforming the parent D–A COF by 127‐fold. Impressively, the catalyst also demonstrated artificial photosynthesis capability, releasing CO at 7.4 µmol g^−1^ h^−1^. To elucidate the underlying pathways of CO_2_ photoreduction and water photooxidation, in situ attenuated total reflection Fourier transform infrared (ATR FT‐IR) spectra was accomplished during the photoreaction cycles. The spectral data provided insights into both redox pathways, including the formation of transient species and key radical intermediates. CO_2_ was shown to adsorb onto Co‐Btt‐Bpy COF and be reduced to *COOH via proton‐coupled electron transfer, followed by dehydrogenation at the Co site to release *CO (Figure 8d). ATR‐FTIR analysis also identified intermediates in the oxygen evolution pathway, outlining a plausible sequence: H_2_O → *H_2_O → *OH → *OOH → *O_2_ → O_2_, where * denotes adsorbed states (Figure 8e). These mechanistic insights were further substantiated by DFT calculations, reinforcing the proposed catalytic cycle (Figure 8f,g). In another contribution, Lu and colleagues showcased an approach integrating the CO_2_ photoreduction and water photo‐oxidation half‐reactions in a single catalytic system.^[^ ^116^ ^]^ A series of crystalline porphyrin‐tetrathiafulvalene COFs, namely TTCOFs, was developed. Under visible light (420–800 nm) and pure CO_2_ (1.0 atm, 298 K) in water, TTCOF‐Zn catalyzed CO generation with remarkable activity (12.33 mmol) and near‐perfect selectivity (∼100%) over 60 h without requiring any sacrificial agents or external photosensitizers. Intriguingly, O_2_ evolution was also observed in the same system, as verified through ^18^O‐labeling. The high performance of the system stems from the electron‐deficient metalloporphyrin complex moieties dominating the LUMO for promoting the CO_2_ reduction, while the electron‐rich tetrathiafulvalene units drive the photoinduced electron transfer process. Yang et al. developed a multifunctional artificial photosynthetic system integrating CO_2_ reduction, H_2_O oxidation, and CO_2_ enrichment within a single catalytic architecture.^[^ ^117^ ^]^ By confining ionic liquids ([Emim]BF_4_) as CO_2_‐enriching agents into the pores of a triazine‐core Zn‐Salen‐based COF, the system achieved a high CO_2_‐to‐CO conversion rate of 105.88 µmol g^−1^ h^−1^ under visible light and 126.51 µmol g^−1^ in 5 h under natural sunlight with 15% CO_2_. Experiments and DFT calculations indicated that the ionic liquids enrich local CO_2_ concentration, while triazine units enhance both oxidation and reduction activities, enabling efficient diluted CO_2_ photoreduction.

Vinylene linkages endow COFs with extended delocalization and lower charge‐transfer resistance, giving them a clear edge over imine‐based frameworks, whose polarized C═N bonds hinder carrier mobility. Zhang and co‐workers introduced two crystalline vinylene‐linked metal‐covalent organic frameworks (MCOFs), VL‐MCOF‐1 and VL‐MCOF‐2, by reticulating Cu_3_(PyCA)_3_ with either a cyano‐substituted (DCTMP) or triazine‐based (TMTA) linker (Figure 8h).^[^ ^118^ ^]^ The electron‐deficient cyano functionality in VL‐MCOF‐1 imparted superior electronic properties, including stronger light absorption, lower bandgap energy (1.93 eV), and faster electron mobility compared to VL‐MCOF‐2. While their CO_2_ uptake capacities were nearly identical, VL‐MCOF‐1 displayed markedly higher catalytic activity for CO_2_ photoreduction, yielding formic acid at a rate nearly 9 and 15 times greater than g‐C_34_N_6_‐COF and the Cu_3_(PyCA)_3_ linker, respectively (Figure 8i). Mechanistic insights confirmed that electrons photogenerated on Cu centers migrated to the cyano/triazine groups, enabling CO_2_ activation and reduction through *OCHO intermediates (Figure 8j). Li et al. synthesized a vinylene‐linked MCOF (UJN‐1) integrating D–π–A characteristics and Cu‐cyclic trinuclear units (Cu–CTUs), yielding exceptional photocatalytic CO_2_ reduction activity.^[^ ^119^ ^]^ The vinylene linkages promoted π‐delocalization, and the D–π–A structure enhanced charge separation. Electron‐deficient, mixed‐valence Cu–CTUs provided dual coordination environments, enabling efficient CO_2_ binding and activation. Experimental and theoretical studies substantiated directional charge migration from triphenylamine donors to Cu–CTU acceptors. Consequently, UJN‐1 achieved a CO production rate of 114.8 µmol g^−1^ with 95% selectivity, surpassing the performance of its imine‐linked counterpart UJN‐2.

Integrating heterojunction units through covalent attachment to the COF backbone is an effective approach to boost CO_2_ photoreduction as these tailored components offer optimized band alignment, redox potential, and catalytic centers. Lin et al. showcased a molecular end‐capping strategy that enhances charge carrier mobility in COFs by grafting organic semiconductors—specifically polycyclic aromatic hydrocarbons—onto the framework, forming an efficient heterojunction interface.^[^ ^120^ ^]^ In particular, the functionalization of TMBen COF with perylene yielded a type‐II band alignment with reduced exciton binding energy, where photogenerated charges are spatially separated between the COF and perylene domains, thereby significantly improving CO_2_ conversion efficiency. The TMBen–Perylene hybrid system demonstrated excellent photocatalytic activity, with CO output increased eightfold compared to the unmodified COF, attributed to suppressed electron–hole recombination and enhanced charge delocalization across the interface. Li et al. developed an alkyl‐linked TiO_2_–Cu‐porphyrin COF heterostructure (TiO_2_@CuPorTT‐COF), where the alkyl bridge plays a key role in directing photogenerated electrons from TiO_2_ to Cu‐porphyrin sites, thereby improving charge separation and boosting photocatalytic CO_2_ reduction with a 5‐fold enhancement in CO evolution than the unbridged counterpart and a quantum efficiency of 0.455% at 380 nm.^[^ ^121^ ^]^ Li et al. constructed hollow core–shell heterojunctions by in situ growth of ZnIn_2_S_4_ on nitrogen‐rich COF nanospheres, enabling efficient CO_2_ adsorption, activation, and charge separation, resulting in a high CO yield of 2895.94 µmol g^−1^ with 95.75% selectivity.^[^ ^122^ ^]^ The Z‐scheme configuration promotes efficient charge separation and directional electron flow, retaining strong redox potentials by mimicking natural photosynthesis. It enables simultaneous utilization of high‐energy electrons and holes, boosting both reduction and oxidation capabilities. A study by Wang et al. demonstrated a porphyrin‐based COF–TiO_2_ hybrid connected via isonicotinic acid to form a Z‐scheme photocatalyst, where the porphyrin unit enhanced light absorption and the Z‐scheme architecture significantly improved charge separation, leading to nearly 10‐ to 25‐fold increased CO_2_ reduction activity compared to individual components.^[^ ^123^ ^]^

Structural engineering of COFs continues to unlock new levels of efficiency in CO_2_ photoreduction. For instance, postsynthetic π‐extension of 3D COFs via annulation of hexaphenyl‐triphenylene to hexabenzo‐trinaphthylene enhanced visible light utilization and CO output by 2.5‐fold.^[^ ^124^ ^]^ Multifunctional integration, such as N‐rich organic cages (enhancing CO_2_ capture), porphyrin light absorbers, and dual Co^2+^/Ni^2+^ active sites, has also led to notable improvements in CO evolution.^[^ ^125^ ^]^ A study by Lan et al. highlights the unique advantage of dual metallosalen‐based COFs, with ZnZn‐Salen‐COF achieving a ∼6‐fold enhancement in CO_2_‐to‐CO conversion rate over its single‐metal analogue under pure CO_2_, and delivering a record‐high activity of 102.1 µmol g^−1^ h^−1^ under simulated flue gas (15% CO_2_).^[^ ^126^ ^]^ In another contribution, a trimetallic 2D COF design featuring Ru, Co, and Cu centers enabled site‐specific enhancements: Ru enhanced optical response, Co facilitated CO generation, and Cu centers enabled selective CH_4_ production.^[^ ^127^ ^]^ Importantly, recent work has shown that decoupling interlayer interactions in 2D COFs substantially improves charge separation, underscoring the importance of interlayer dynamics and offering a new direction for efficiency improvement.^[^ ^128^ ^]^

### 4.2 Redox‐Active Frameworks: MOFs and COFs in Electrocatalytic CO_2_ Reduction

**Figure 9:**
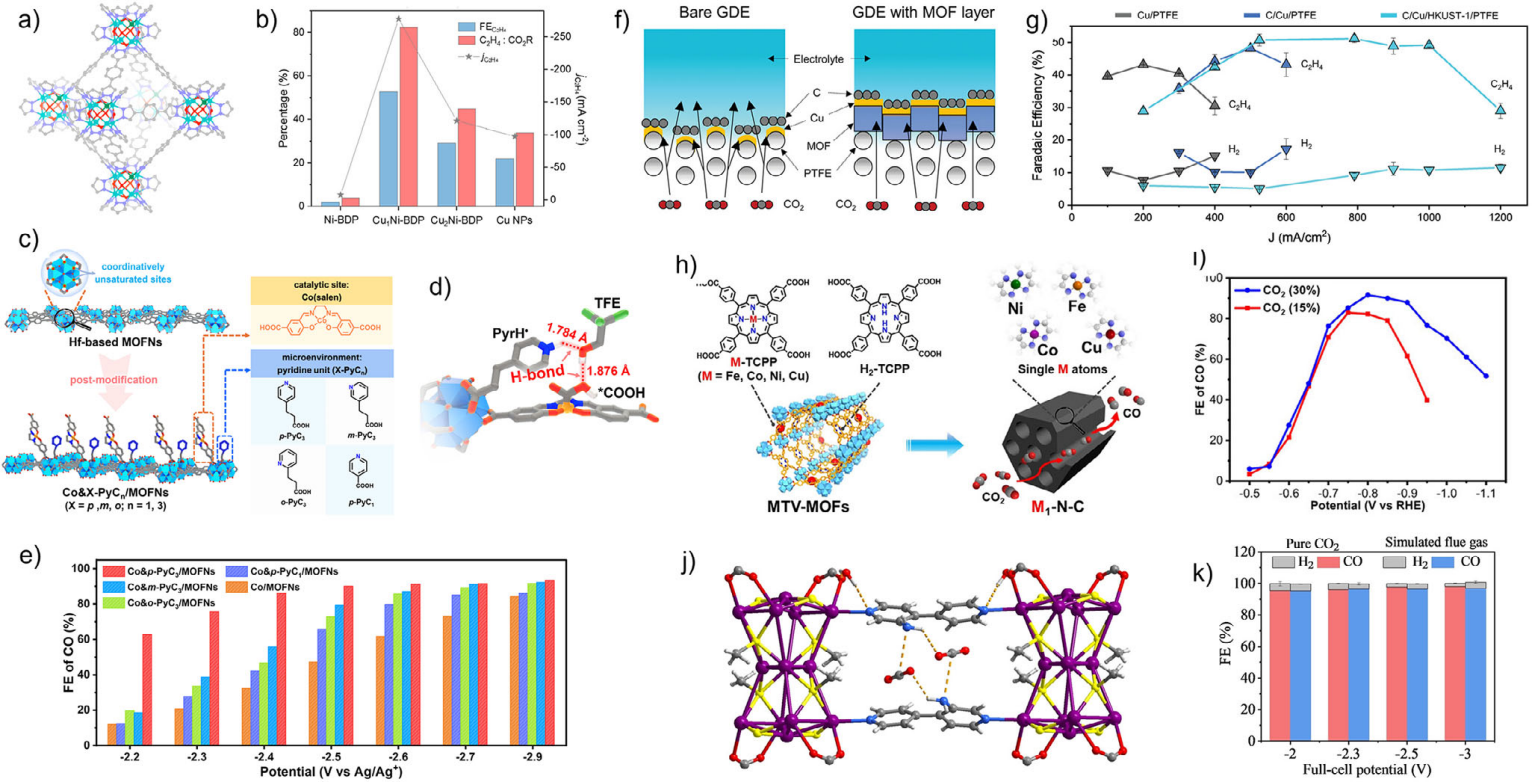
a) Depiction of the porous cage architecture in Cu_1_Ni‐BDP MOF. b) FE, product distribution ratio, and C_2_H_4_ partial current density (*j*_C2H4_) for the Ni‐BDP MOF, Cu_1_Ni‐BDP MOF, Cu_2_Ni‐BDP MOF, and Cu NPs under flow cell conditions at −1.3 V versus RHE. Adapted with permission from ref. [131] Copyright 2023 American Chemical Society. c) Synthetic route and coordination structure of Co&X‐PyC*_n_*‐modified MOF nanosheets (X = *o*, *m*, *p* refers to the pyridine N position; n = alkyl chain length). d) Depiction of the hydrogen‐bond‐stabilized *COOH⋯TFE⋯PyrH^·^ species. e) Comparison of CO FEs for Co&X‐PyCn/MOFNs. Adapted with permission from ref. [132] Copyright 2025 the Author(s). Published by PNAS. f) Schematic side‐view of unmodified and MOF‐integrated electrodes in CO_2_RR flow cell. g) C_2_H_4_ and H_2_ selectivities of various electrodes with increasing current density in 1 m KOH. Adapted with permission from ref. [138] Copyright 2022 Wiley‐VCH GmbH. h) Strategy for constructing M_1_‐N‐C single‐atom catalysts derived from multivariate MOFs for CO_2_RR. i) FE for CO production over Ni_1_‐N‐C in KHCO_3_ with 30% and 15% CO_2_ feed. Adapted with permission from ref. [137] Copyright 2020 Wiley‐VCH GmbH. j) Binding sites for CO_2_ and H_2_O identified on Ag_12_‐bpy–NH_2_. k) CO selectivity of Ag_12_‐bpy–NH_2_ in CO_2_ and simulated flue gas. Adapted with permission from ref. [139] Copyright 2023 Wiley‐VCH GmbH.

*MOFs*: MOFs have emerged as a versatile class of electrocatalysts for CO_2_RR, offering a highly tunable platform for optimizing activity, selectivity, and operational stability. Recent advances highlight several key strategies in MOF design to address the challenges in CO_2_RR. Innovative coordination environments within MOFs have enabled stabilization of reactive metal centers and precise control of catalytic function. For example, vertically conductive 2D‐*vc*‐MOFs, such as triptycene‐based Cu‐MOFs, exhibit enhanced catalytic activity due to reduced interlayer interactions and improved site accessibility.^[^ ^129^ ^]^ Incorporating stable Cu(I) clusters with strong cuprophilic interactions, as seen in NNU‐50, effectively prevents reduction‐induced deactivation, yielding high methane selectivity and current density.^[^ ^130^ ^]^ Leveraging asymmetric Ni and Cu active sites in a pyrazolate‐linked scaffold, bimetallic MOFs like Cu_1_Ni‐BDP (Figure 9a) attained high ethylene selectivity at current densities approaching industrial practice (Figure 9b).^[^ ^131^ ^]^ The catalytic microenvironment within MOFs can be precisely tuned through ligand design and post‐synthetic modifications. In a study by Jiang et al., Hf‐MOF nanosheets were post‐synthetically decorated with Co(salen) and pyridyl‐substituted alkyl carboxylic acids to tune local coordination (Figure 9c).^[^ ^132^ ^]^ 3‐(Pyridin‐4‐yl)propionic acid, featuring a para‐positioned N atom, most significantly enhanced CO_2_ electroreduction (Figure 9e) by forming an in situ pyridinyl radical that stabilizes the *COOH intermediate via hydrogen bonding (Figure 9d), reducing its energy barrier. Similarly, the use of pyrazolate bridges,^[^ ^131^ ^]^ Bi─N coordination,^[^ ^133^ ^]^ or [Zr_48_Ni_6_] cages^[^ ^134^ ^]^ introduces electron density modulation or confinement effects that promote multielectron transfer and stabilize transition states during CO_2_RR.

MOFs have also been integrated with other materials to create hybrid systems that overcome conductivity limitations and boost catalytic performance. Surface modification of Cu with Zr‐based metal–organic layers (MOLs) induces in situ nano‐structuring of the catalyst during operation, enhancing CH_4_ selectivity.^[^ ^135^ ^]^ MOF coatings on Cu_2_O nanocrystals have been used to modulate product distributions, e.g., favoring syngas formation by controlling facet exposure and diffusion barriers.^[^ ^136^ ^]^ To move CO_2_RR closer to practical deployment, several studies have explored MOF‐based systems under low CO_2_ concentrations. Jiang et al. synthesized pyrolyzed porphyrinic MOFs (M_1_‐N‐C, M = Fe, Co, Ni, Cu) that serve as model single‐atom catalysts (Figure 9h), showing that Ni_1_‐N‐C achieves outstanding CO selectivity even at 15% CO_2_ (Figure 9i).^[^ ^137^ ^]^ Likewise, MOF‐functionalized gas diffusion electrodes (GDEs) (Figure 9f) enhance local CO_2_ concentration and phase boundary control, enabling high‐rate ethylene production (Figure 9g) in flow cells and MEA devices—reaching 49% FE at 1 A cm^−2^, which surpasses the technoeconomic benchmark of 200 mA cm^−2^ current density for industrially relevant CO_2_RR.^[^ ^138^ ^]^ A significant milestone was achieved with Ag_12_bpy‐NH_2_, a MOF capable of capturing CO_2_ from simulated flue gas (Figure 9j) and directly converting it electrochemically to CO with a FE of 96% (Figure 9k) and operational durability over 300 h.^[^ ^139^ ^]^ This material successfully bridges the CO_2_ capture and reduction processes, representing a practical step toward integrated carbon management technologies. In summary, MOFs offer a highly adaptable platform for electrocatalytic CO_2_RR through rational design of active sites, manipulation of local environments, and hybrid integration. These advances not only improve performance under pure CO_2_ but also enable efficient catalysis under realistic, low‐concentration conditions, pushing MOFs toward industrial relevance.

*COFs*: COFs offer distinct advantages as electrocatalysts for CO_2_ reduction due to their highly ordered and tunable porous architectures, which enable precise spatial arrangement of active sites, facilitate mass transport, and allow for modular functionalization. Their extended π‐conjugation networks promote efficient charge transport, while the incorporation of redox‐active metal centers into the framework can enhance catalytic activity and selectivity. Moreover, COFs provide a robust platform for integrating multiple functionalities and tailoring the local chemical environment around catalytic sites.

**Figure 10:**
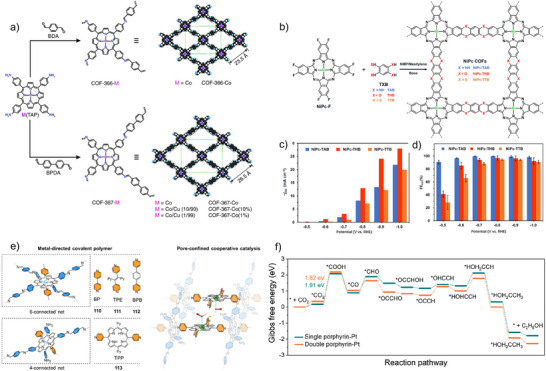
a) Schematic depiction of the construction of metalloporphyrin‐based 2D COFs. Reprinted with permission from ref. [140] Copyright 2015 American Association for the Advancement of Science. b) Construction scheme for three Ni‐phthalocyanine COFs incorporating three different types of linkages: piperazine, dioxin, and dithiine. c) CO current density for the three COFs across varying potentials. d) CO Faradaic efficiency for the three COFs across varying potentials. Adapted with permission from ref. [145] Copyright Creative Commons Attribution 4.0 International license. e) Schematic of MOCOF synthesis via metal‐coordination and imine linkage; pore‐confined cooperative catalysis of twin metalloporphyrin units. f) Calculated free energy pathways for CO_2_‐to‐ethanol conversion on single porphyrin‐Pt sites and twin porphyrin‐Pt sites within Pt‐MOCOF. Adapted with permission from ref. [148] Copyright 2025 American Chemical Society.

Yaghi and co‐workers pioneered the use of cobalt‐porphyrin‐containing COFs for electrocatalytic CO_2_‐to‐CO conversion in aqueous media (Figure 10a).^[^ ^140^ ^]^ COF‐366‐Co exhibited high CO selectivity and a FE of 90% at −0.67 V, far exceeding that of the Co‐porphyrin monomer. By expanding the lattice (replacing BDA with BPDA linker in COF‐367‐Co), the turnover number of electroactive cobalt sites (TON_EA_) was increased from ∼34000 to ∼48000. Partial replacement of Co‐porphyrin with Cu‐porphyrin led to diluted active site architectures (COF‐367‐Co 10% and 1%), significantly boosting the TOF_EA_ of individual cobalt centers—COF‐367‐Co (1%) achieved a TON_EA_ of ∼296000. COF‐367‐Co thin films outperformed the powder form under identical electrochemical conditions, demonstrating the benefit of film‐based architectures for charge transport and catalyst accessibility. In a separate investigation, the same group introduced a strategy to modulate the electrocatalytic performance of COF‐366‐Co by altering the functional groups on the BDA linker.^[^ ^141^ ^]^ Variants such as COF‐366‐(OMe)_2_‐Co, COF‐366‐F‐Co, and COF‐366‐(F)_4_‐Co were synthesized, revealing that even peripheral modifications—outside the porphyrin units—can significantly impact CO_2_ reduction activity. This underscores the potential of the COF framework itself to actively influence catalysis beyond direct modification of the metal sites.

Leveraging the molecular CO_2_ reduction competency of metallophthalocyanines, these tetrapyrrole analogues were integrated into 2D COFs. In a contribution by Han et al., the unique advantage lies in the integration of polyimide‐linked phthalocyanine COFs into cathodes, achieving exceptional CO selectivity (FE up to 97%) and outstanding long‐term performance with a TON of 277000 and TOF of 2.2 s^−1^ over 40 h—highlighting both high activity and durability.^[^ ^142^ ^]^ Another work by Yue et al. introduced robust dioxin‐linked bimetallic phthalocyanine COFs featuring ordered 1D π‐columns for rapid electron transport, achieving record‐high CO FE (97%) and TOF (2.87 s^−1^) for COF‐based electrocatalytic CO_2_ reduction, with excellent stability and low overpotential.^[^ ^143^ ^]^ A recent study presented a Co‐phthalocyanine COF (PEH‐COF) with COOH/OH‐functionalized catalytic cages that modulate the local microenvironment of Co sites, where stabilized hydrated K^+^ ions enhance proton‐coupled electron transfer and lower energy barriers, enabling efficient and durable CO_2_‐to‐CH_3_OH electrocatalysis.^[^ ^144^ ^]^ In a notable work by Chen et al., three distinct Ni‐phthalocyanine COFs varying in their linkage chemistries—dioxin, piperazine, and dithiine—were constructed (Figure 10b).^[^ ^145^ ^]^ Remarkably, even a subtle alteration in the linkage type markedly altered the catalytic behavior. The dioxin‐linked COF showed superior electrocatalytic activity with a CO current density of −27.99 mA cm^−2^ at −1.0 V versus RHE (Figure 10c), while the piperazine‐linked variant excelled in selectivity, reaching a CO Faradaic efficiency of 90.7% at an exceptionally low overpotential of 0.39 V (Figure 10d). Computational analyses suggested that the dioxin linkage facilitated stronger stabilization of the *COOH intermediate, which contributed to its enhanced reactivity.

COF nanosheets possess ultrathin, layered structures with high surface area‐to‐volume ratios, enabling more accessible exposure of active sites and faster mass/electron transport. Their shortened out‐of‐plane charge pathways facilitate efficient charge separation and transfer, while their structural tunability allows precise incorporation of catalytic moieties, making them ideal candidates for efficient and selective CO_2_ electroreduction. Zhu et al. designed a series of metalloporphyrin‐tetrathiafulvalene COFs (M‐TTCOFs) where tetrathiafulvalene acts as an internal electron donor/carrier to promote directional electron transfer to metalloporphyrin centers, enabling high CO selectivity (FE_CO_ up to 91.3%) and durability.^[^ ^146^ ^]^ Upon exfoliation into ∼5 nm nanosheets, Co‐TTCOFs exhibited exceptional performance, achieving nearly 100% FE_CO_ at −0.8 V, due to increased surface area and enhanced exposure of catalytic sites. Barman and co‐workers employed redox‐active triphenylamine‐based COF nanosheets as a metal‐free electrocatalyst for CO_2_ conversion in a 0.2 M phosphate buffer (pH 7.2).^[^ ^147^ ^]^ Under mild aqueous conditions, the system selectively generated methanol as the sole carbon‐based liquid product with a peak FE of 51.6%. Notably, the processproceeded at a low overpotential of 210 mV, highlighting the catalyst's high efficiency despite the absence of metal centers.

MOF–COF hybrids combine the modularity, crystallinity, and tunable porosity of MOFs with the chemical robustness and structural versatility of COFs. A recent study by Cui et al. introduced the first highly crystalline single‐crystal MOF–COF (MOCOF) hybrids via a one‐pot heteroassembly strategy, demonstrating a novel design principle that integrates reversible coordination and robust covalent self‐assembly (Figure 10e).^[^ ^148^ ^]^ Their structures were ascertained through single‐crystal 3D electron diffraction. The resulting Pt‐MOCOF bears confined metalloporphyrin sites that synergistically capture and activate CO_2_, enabling exceptional photoelectrocatalytic CO_2_‐to‐ethanol conversion with high FE (83.5%), carbon selectivity (91.7%), and long‐term operational stability (100 h with 95% retained activity). DFT calculations revealed that twin‐site metalloporphyrin catalysts offer stronger CO_2_ adsorption and lower activation barriers compared to single‐site models, with enhanced CO_2_ activation, stabilized intermediates, and more favorable energetics for C─C coupling, ultimately facilitating efficient ethanol production (Figure 10f). Table 2 provides a comparative overview of representative NCs, MOFs, and COFs, highlighting their target products, production rates, current densities, TOFs, selectivity/FEs, and stability under photo‐ and electrocatalytic CO_2_ reduction conditions.

**Table 2:** Comparison of representative NCs, MOFs, and COFs for CO_2_ reduction, summarizing target products, activity, selectivity, and stability metrics.

Photocatalytic CO_2_RR					
Metal NCs					
Catalyst	Target product(s)	Yield rate	Selectivity	Stability	Ref.
Au_25_(PET)_18_/gCN	CO	166 × 10^−9^ mol s^−1^ g_Au_^−1^	9.87%	12 h	[57]
Ag_25_(SPhMe_2_)_18_	CH_4_	28.95 µmol h^−1^ mg^−1^	∼100%	10 h	[61]
Ag_24_(Si_2_W_18_O_66_)_3_	HCOOH	4.66 µmol h^−1^	∼90%	5 h	[62]
Cu_6_–NH	CO	24.8 µmol h^−1^ g^−1^	∼100%	24 h	[64]
Co_33_Nb_54_O_188_	CO	27.4 µmol h^−1^	63.6%	1 h × 5	[66]

MOFs					
Catalyst	Target product(s)	Yield rate	Selectivity	Stability	Ref.
Pt/Ni‐MOF	CO/CH_4_	606/135 µmol g^−1^ h^−1^	80% (CO)	10 h × 3	[94]
P@U	HCOOH	146.0 µmol g^−1^ h^−1^	73.2%	25 h	[98]
Fe/Ti‐BPDC	HCOOH	703.9 µmol g^−1^ h^−1^	>99.7%	5 h × 10	[99]
MIL‐100(Fe)	CH_4_	47.34 µmol g^−1^ h^−1^	∼98%	5 h × 7	[101]
g‐C_3_N_4_/UiO‐66(Zr/Ce)	CH_3_OH/C_2_H_5_OH	54.71/38.10 µmol g^−1^ h^−1^	–	4 h × 12	[107]

COFs					
Catalyst	Target product(s)	Yield rate	Selectivity	Stability	Ref.
N_3_‐COF	CH_3_OH	0.571 µmol g^−1^ h^−1^	–	24 h × 7	[109]
Bpy‐sp^2^c‐COF	CO	1040 µmol g^−1^ h^−1^	81%	17.5 h	[112]
Re^I^bpyDBC COF	CO	1160 µmol g^−1^ h^−1^	–	72 h	[113]
Co‐Btt‐Bpy COF	CO	9800 µmol g^−1^ h^−1^	78.7%	4 h × 5	[115]
TTCOF‐Zn	CO	2.055 µmol g^−1^ h^−1^	∼100%	60 h	[116]

Electrocatalytic CO_2_RR						
Metal NCs						
Catalyst	Target product(s)	Yield rate	Current density	FE	Stability	Ref.
Au_25_(SC_2_H_4_Ph)_18_^−^	CO	1.26 mmol cm^−2^ h^−1^ (−1.0 V vs RHE)	–	∼100% (−1.0 V vs RHE)	1 h (−1.0 V vs RHE)	[67]
Pd_1_Au_24_(SR)_18_	CO	–	∼1770 mA mg_metal_^−1^ (−1.2 V vs RHE)	∼100% (−1.2 V vs RHE)	6 h (−0.6 V vs RHE)	[69]
Ag_32_(C≡CAr)_24_	CO	–	9.05 mA cm^−2^ (−1.0 V vs RHE)	96.4% (−0.8 V vs RHE)	15 h (−0.8 V vs RHE)	[80]
Cu_8_(^t^BuS)_4_(L1)_4_	HCOOH	–	–	∼92% (−1.0 V vs RHE)	8 h (−0.9 V vs RHE)	[83]
[Cu_14_(SC_6_H_3_F_2_)_3_(P(PhF)_3_)_8_H_10_]^+^	CH_4_ + C_2_H_4_	–	–	54.3% (−1.2 V vs RHE)	5 h (−0.9 V vs RHE)	[92]

MOFs						
Catalyst	Target product(s)	TOF	Current density	Selectivity	Stability	Ref.
Cu_1_Ni‐BDPMOF	C_2_H_4_	–	278 mA cm^−2^ (−1.3 V vs RHE)	52.7% (−1.3 V vs RHE)	25 h (−1.3 V vs RHE)	[131]
Co&*p*‐PyC_3_ /MOFNs	CO	–	–	>90% (−2.5 V vs Ag/Ag^+^)	5 h (−2.5 V vs Ag/Ag^+^)	[132]
F‐Cu_2_O@ZIF‐8	CO	–	–	83.5% (−1.1 V vs RHE)	–	[136]
Ni_1_‐N‐C	CO	11 315 h^−1^ (−0.8 V vs RHE)	27 mA cm^−2^ (−0.8 V vs RHE)	96.8% (−0.8 V vs RHE)	10 h (−0.8 V vs RHE)	[137]
Ni − N_4_ − C	CO	3488 h^−1^ (−2.3 V; Full‐cell potential)	380 mA cm^−2^ (−3.0 V; Full‐cell potential)	97.3% (−3.0 V; Full‐cell potential)	300 h (−2.3 V; Full‐cell potential)	[139]

COFs						
Catalyst	Target product(s)	TOF	Current density	Selectivity	Stability	Ref.
COF‐367‐Co	CO	165 h^−1^ (−0.67 V vs RHE)	3.3 mA cm^−2^ (−0.67 V vs RHE)	91% (−0.67 V vs RHE)	24 h (−0.67 V vs RHE)	[140]
COF‐366‐Co films	CO	–	45 mA mg_cobalt_^−1^ (−0.67 V vs RHE)	87% (−0.67 V vs RHE)	12 h (−0.67 V vs RHE)	[141]
CoPc‐PI‐COF‐1	CO	4.9 s^−1^ (−0.9 V vs RHE)	21.2 mA cm^−2^ (−0.9 V vs RHE)	95% (−0.9 V vs RHE)	40 h (−0.7 V vs RHE)	[142]
PEH‐COF	CH_3_OH	–	100.4 mA cm^−2^ (−1.3 V vs RHE)	37% (−1.3 V vs RHE)	9 h (−1.3 V vs RHE)	[144]
NiPc‐TAB	CO	–	8.2 mA cm^−2^ (−0.8 V vs RHE)	99.90% (−0.8 V vs RHE)	10 h (−0.8 V vs RHE)	[145]

## 5 Gaps, Targets, and Promising Avenues

### 5.1 Mechanistic Comprehension and Strategic Design

**Gaps**:

- Current understanding of CO_2_ activation and reduction mechanisms in heterogeneous photo/electrocatalysts, such as MOFs and COFs, remains inadequate. Many studies often transpose mechanisms from homogeneous catalysis, overlooking the complexities unique to solid‐state systems, including structural and electronic heterogeneity of active sites, structural defects, interfacial charge separation and transport, and diffusion limitations arising from confined pore architectures.
- The kinetics and thermodynamics governing photo‐ and electro‐induced charge separation and transfer in MOFs, COFs, and metal NCs remain poorly resolved at the atomic level. In MOFs and COFs, charge carrier generation and migration are influenced by factors such as linker conjugation, metal node redox properties, framework connectivity, crystallinity, and the presence of structural defects, while interfacial charge transfer depends critically on the alignment of energy levels with substrates and the accessibility of active sites through the pore network. In contrast, in NCs, charge carrier dynamics are governed by quantum confinement effects, core–ligand interactions, surface atom coordination, and electronic shell structures, which collectively dictate their light absorption, redox behavior, and charge transfer to adsorbed CO_2_ intermediates. However, direct experimental evidence elucidating these processes is limited, often due to the lack of *operando* characterization techniques with sufficient spatial and temporal resolution.
- In NCs, the reaction pathways for CO_2_ activation are often oversimplified, with insufficient mechanistic clarity regarding the roles of ligand environments, electronic shell closure, and surface charge distributions. Ligands not only stabilize the cluster core but also modulate its electronic structure, influencing the adsorption strength and orientation of CO_2_ molecules and key intermediates. For instance, Bootharaju et al. showed that ligand shells on Ag_25_ NCs create localized hydrophobic microenvironments which dramatically alter intermediate binding (e.g., *CO) and boost CO formation—with operando attenuated total reflection–surface‐enhanced infrared absorption spectroscopy (ATR‐SEIRAS) and theory revealing ligand‐induced modulation of active‐site behavior beyond core‐only models.^[^ ^149^ ^]^ Electronic shell closure, governed by the discrete energy levels of atomically precise clusters, affects redox potentials and charge delocalization, which are critical for multielectron transfer steps. Additionally, nonuniform surface charge distributions—arising from asymmetric coordination environments or anisotropic ligand binding—can create localized electrostatic fields that alter CO_2_ binding modes and activation barriers. These factors collectively contribute to reactivity but are rarely captured in simplified models or routinely addressed in experimental studies.
- A major unresolved challenge lies in controlling product selectivity during photoelectrochemical CO_2_RR, which hinges on the competition between proton‐coupled electron transfer (PCET) steps leading to either C_1_ (CO, HCOOH, CH_4_) or C_2+_ (C_2_H_4_, ethanol) products. The selectivity is governed by several intertwined factors: i) the binding strength and orientation of key intermediates (*COOH, *CO, *CHO, COCOH), which dictate whether C─H protonation or C─C coupling dominates; ii) the local proton environment and water activity, which modulate PCET kinetics and favor specific hydrogenation pathways; iii) the alignment of catalyst energy levels with CO_2_ reduction and competing HER, which determines whether electrons are funneled into productive CO_2_RR channels; iv) the spatial confinement and hydrophobic/hydrophilic balance within MOF/COF pores, which influence intermediate stabilization and diffusion; and (v) the temporal dynamics of photogenerated charge carriers, where lifetime, separation efficiency, and recombination rates strongly bias product distribution. For example, extended π‐conjugation in COFs can prolong exciton lifetimes and enhance C─C coupling probability, while in Cu‐based NCs, the stabilization of COCOH by lattice hydrides has been shown to be decisive for C_2+_ formation. These mechanistic determinants are often system‐specific but converge on a central theme: selectivity emerges from a delicate interplay of electronic, structural, and interfacial factors that remain only partially understood.

**Targets**:

- An essential goal is to elucidate reaction intermediates and transition states through in situ and *operando* characterization techniques, e.g., ambient‐pressure X‐ray photoelectron spectroscopy (AP‐XPS), time‐resolved infrared and Raman spectroscopy, and ultrafast transient absorption spectroscopy. These techniques will enable real‐time tracking of adsorbed species, identification of short‐lived intermediates, and mapping of energy landscapes under working conditions, thereby bridging the gap between surface chemistry and catalytic function in photo‐ and electrocatalytic CO_2_ reduction.
- Another important objective is to apply theoretical frameworks, such as time‐dependent density functional theory (TD‐DFT) and nonadiabatic molecular dynamics, in combination with machine learning (ML) algorithms, to model charge carrier dynamics, interfacial electron transfer, and band edge alignments in complex heterogeneous systems. This integrated approach aims to accelerate the prediction of key descriptors governing activity and selectivity, while also guiding the rational design of catalysts with tailored electronic structures and reaction energetics.
- A critical target is to decode the relationship between the electronic effects of NCs—including quantum confinement, d‐band center tuning, and surface lattice strain—and their catalytic selectivity toward C_1_ (e.g., CO, HCOOH, CH_4_) and C_2_ (e.g., C_2_H_4_, ethanol) products. Understanding how atomic‐scale structural motifs and electronic configurations govern binding energies, charge localization, and activation barriers will enable precise tuning of NC properties for enhanced product specificity.
- A further target is to systematically map how external parameters—such as applied potential, illumination wavelength/intensity, electrolyte composition, and pH—reshape the energy landscape of CO_2_RR and shift the balance between C_1_ and C_2_^+^ pathways. Developing predictive models that integrate these extrinsic factors with intrinsic material descriptors will provide a holistic framework for steering product selectivity. In particular, coupling operando spectroscopy with microkinetic modeling could reveal potential‐dependent branching ratios of intermediates and clarify how reaction conditions translate into selective product outcomes.

**Promising Avenues**:

- One promising direction involves engineering catalysts with tunable coordination environments that can dynamically respond to changes in light or electrochemical potential. This approach involves using stimuli‐responsive ligands or flexible organic linkers in MOFs/COFs or surface‐bound moieties on metal nanoclusters that actively modulate the local environment around active sites. Such adaptability could enhance selectivity and activity by enabling real‐time conformational or electronic tuning during catalysis.
- The integration of single‐particle spectroscopies and electrochemical scanning probe techniques—such as tip‐enhanced Raman spectroscopy (TERS), photoconductive atomic force microscopy (pc‐AFM), and scanning electrochemical cell microscopy (SECCM)—offers a powerful route to spatially resolving photocatalytic activity at the nanoscale. These high‐resolution methods can reveal heterogeneities in charge transport, local catalytic behavior, and reaction kinetics across individual catalyst particles or domains, uncovering performance‐limiting features that are averaged out in ensemble measurements.
- Systematically constructing libraries of photo‐ and electrocatalytic materials by varying key molecular components—such as ligands, metal nodes, and π‐conjugated linkers—offers a promising route to rapidly identify and optimize catalytic performance. Using high‐throughput synthesis and characterization approaches, such libraries can probe how molecular structure, coordination environment, and electronic delocalization influence light absorption, exciton dissociation, and product selectivity. Integrating these datasets with ML tools will further enable predictive structure–function mapping and rapid identification of high‐performance catalyst formulations.
- An emerging avenue is the deliberate engineering of catalytic microenvironments to bias specific product channels. For example, hydrophobic pore walls in COFs can suppress competing HER while concentrating CO_2_ near active sites, favoring C_1_ products, whereas metal–nitrogen coordination motifs in COFs and MOFs can stabilize CO intermediates long enough to undergo C─C coupling. Similarly, site‐isolated single‐atom dopants within NCs or frameworks can introduce asymmetric charge distributions that preferentially stabilize certain intermediates. These strategies, coupled with rational modulation of light‐harvesting and charge‐transfer pathways, may ultimately enable predictive and controllable synthesis of CO_2_‐derived fuels and chemicals.

### 5.2 Materials Engineering for Performance Optimization

**Gaps**:

- The design of MOF‐ and COF‐based photocatalysts for CO_2_ reduction frequently relies on sacrificial electron donors or acceptors to facilitate charge separation and suppress recombination. Although this approach is effective under laboratory conditions, it fails to replicate the redox‐neutral environment required for practical CO_2_ conversion systems. This reliance limits the applicability of these materials for real‐world CO_2_ reduction, where bias‐free operation and long‐term stability are essential. A key challenge remains the integration of co‐catalysts or redox mediators that can sustain charge separation under realistic, sacrificial agent‐free conditions.
- NC‐based catalysts are prone to agglomeration and structural degradation under photocatalytic and electrocatalytic conditions, primarily due to their high surface energy, labile ligand shells, and small nuclearity. Upon light irradiation or applied bias, destabilizing factors such as photoinduced desorption of ligands, oxidative/reductive surface transformations, and interactions with reactive intermediates (e.g., CO_2_˙^−^, H˙) can trigger irreversible cluster growth or sintering. These processes lead to a loss of atomic precision, diminished active site accessibility, and the emergence of ill‐defined or inactive aggregates. Furthermore, their poor interfacial adhesion to support can result in leaching or detachment, further undermining catalyst recyclability and long‐term performance. These stability issues remain a critical bottleneck in deploying NCs in *operando* CO_2_ conversion systems.
- The lack of directional growth and facet engineering in crystalline MOFs and COFs hinders the exposure of catalytically active facets and limits anisotropic charge transport. Most MOFs and COFs are synthesized as isotropic or randomly oriented microcrystals, which can trap photogenerated carriers within the bulk or reduce accessibility to reactive edge or surface sites. Enhancing control over crystal orientation, facet‐selective growth, and preferential stacking could significantly improve light harvesting, charge mobility, and interfacial reaction kinetics.

**Targets**:

- An important objective is to replace sacrificial reagents with built‐in donor–acceptor motifs in MOFs and COFs, such as combining electron‐rich linkers with photoactive metal nodes or redox‐active organic units. Enhancing internal electric fields through judicious linker dipole alignment or framework polarization can facilitate efficient charge separation and directional carrier transport, enabling unbiased CO_2_ photoreduction under solar illumination.
- Another goal is to stabilize metal NCs by employing strategies such as covalent anchoring to functionalized framework sites or encapsulation within the cavities of MOFs or COFs.^[^ ^150^, ^151^, ^152^, ^153^ ^]^ Such strategies can suppress agglomeration, inhibit surface reconstruction, and preserve the atomically precise structure of the clusters under operational conditions, ensuring sustained catalytic activity and selectivity over prolonged cycles.
- Achieving directional charge transport is critical for efficient CO_2_ photoreduction and can be realized through the design of MOF and COF structures with anisotropic crystal habits or oriented channels that align with the direction of photogenerated charge carrier flow. Structural tuning via controlled facet orientation, ordered π‐stacking, or axial conjugation pathways can promote carrier mobility, mitigate recombination, and improve access to catalytically active sites, translating into greater photocatalytic efficiency and product selectivity.

**Promising Avenues**:

- The development of 2D MOF and COF nanosheets or thin films represents a promising frontier, offering large surface‐to‐volume ratios, reduced charge transport distances, and superior accessibility of active sites. These ultrathin platforms provide intimate contact with conductive substrates, enabling efficient electrochemical interfaces and rapid charge carrier extraction. Their planar morphology also allows better control over layer orientation and stacking, which can be tuned to optimize charge mobility, suppress recombination, and boost light absorption in photocatalytic and electrocatalytic CO_2_ reduction systems.
- An impactful approach involves the development of 3D hierarchical architectures that integrate mesoporous/macroporous channels into crystalline MOF or COF matrices. These multiscale porous structures combine extensive surface area with enhanced mass transport, facilitating faster reactant diffusion and product desorption. Simultaneously, the increased optical path length and scattering within such architectures enhance light‐harvesting efficiency. This structural integration of nanoscale order with microscale porosity elevates both catalytic turnover and photonic efficiency, rendering them ideal for solar‐to‐chemical energy conversion.
- Incorporating plasmonic nanostructures, like Au or Ag NCs, into MOFs or COFs offers an effective route to achieve visible‐light sensitization through plasmonic enhancement mechanisms.^[^ ^154^ ^]^ These include hot‐electron injection from the plasmonic metal into the conduction band of the framework, as well as localized surface plasmon resonance (LSPR)‐induced near‐field amplification that boosts light absorption in the surrounding semiconductor. Such hybrid systems not only extend the photocatalytic response into the visible and near‐infrared regions but also promote rapid carrier generation and interfacial charge transfer.

### 5.3 Targeting Selective, Scalable, and Sustainable Catalysis

**Gaps**:

- Poor selectivity toward higher‐value multicarbon (C_2+_) products such as ethylene, ethanol, or acetate remains a pressing issue, as most systems predominantly yield C_1_ species. This is primarily owing to inadequate control over C─C coupling steps and unstable intermediate binding configurations.
- A key challenge lies in the inadequate tunability in intermediate binding of current reticular and cluster‐based catalysts, which often lack the electronic and geometric precision required to stabilize specific CO_2_ reduction intermediates (e.g., *CO, *CHO, *COH) in a pathway‐selective manner, restricting mechanistic control over product formation.
- Despite promising lab‐scale performance, the transition of MOF‐ and COF‐based systems to real‐world CO_2_ conversion devices is hindered by unresolved issues in scalability and operational stability. Continuous illumination or applied electrochemical bias often induces degradation phenomena due to photo‐instability, framework collapse, or metal leaching.

**Targets**:

- A major design goal is to engineer multifunctional active sites capable of concurrently facilitating CO_2_ adsorption, activation, and efficient electron transfer, thereby lowering kinetic barriers in the initial reduction steps. Heterometallic NCs, in which different metal centers synergize to stabilize intermediates and mediate redox steps, offer a promising solution. Analogously, introducing heteroatoms or secondary metal sites into porous frameworks can enhance local charge density, modulate the electronic structure of the framework, and foster cooperative catalytic functions.
- Advancing selective electrocatalytic CO_2_ conversion hinges on developing electrocatalysts with tailored electrochemical properties, including high electrochemical surface area (ECSA) to maximize exposed active sites, enhanced intrinsic conductivity to ensure rapid electron transport, and redox‐active centers with suitable binding energies for selective CO_2_‐to‐C_1_/C_2_ product formation. This requires precise control over morphology (e.g., thin films, nanosheets, nanowires), electronic structure (e.g., ligand field tuning or π‐conjugation), and local environment (e.g., proton relays, hydrophobicity) to steer reaction pathways and suppress competing pathways like hydrogen evolution.
- To speed up the discovery pipeline for CO_2_ reduction catalysts, it is vital to implement high‐throughput experimental platforms that can rapidly evaluate catalytic performance across material libraries. Tools such as electrochemical microarrays, automated potentiostatic scanning systems, or photoelectrochemical microreactors enable parallel testing of compositional libraries or structural variants under controlled conditions. Coupled with *operando* spectroscopy, data analytics, and ML, these platforms can rapidly uncover structure–activity relationships, and support rapid iteration in catalyst design for CO_2_ conversion.

**Promising Avenues**:

- A compelling direction involves fabricating in situ‐grown MOF/COF films on conductive substrates with controlled orientation and strong electronic coupling to electrodes. Such configurations facilitate rapid charge injection by minimizing interfacial impedance and ensuring intimate contact between the catalytic material and current collector. Oriented growth—particularly along crystallographic axes aligned with π‐conjugated pathways or charge transport channels—can substantially improve carrier mobility and lower recombination rates. Techniques like interfacial crystallization, solvothermal solvothermal film growth, or layer‐by‐layer assembly enable precise control over film thickness, crystallinity, and alignment, enabling better integration into scalable catalytic modules for CO_2_ conversion.
- The development of intrinsically conductive COFs can be realized through the integration of extended π‐conjugated backbones, redox‐active moieties such as porphyrins, quinones, or phthalocyanines, and molecular dopants. These design features can transform COFs from insulating to semiconducting or even metallic regimes, enabling efficient intra‐framework charge transport without the need for external additives. Recent advances illustrate effective strategies to harness these properties for CO_2_RR. For instance, a phthalocyanine‐based conjugated COF demonstrated that extended conjugation can deliver high conductivity while maintaining open channels for CO_2_ diffusion, thereby enabling efficient CO_2_‐to‐CO conversion.^[^ ^155^ ^]^ Likewise, conductive 2D NiPc‐based MCOFs featuring metal tetraaza[14]annulene linkages showcased how in‐plane d–π conjugation can simultaneously enhance charge transport and framework stability, leading to improved CO_2_RR reduction activity.^[^ ^156^ ^]^ Building on this principle, ladder‐type π‐conjugated COFs with rigid architectures have been developed, in which extended electronic delocalization facilitates efficient CO_2_RR catalysis.^[^ ^157^ ^]^ In parallel, donor–acceptor (D–A) engineered COFs—such as cobalt‐porphyrin frameworks incorporating alternating donor (e.g., benzodithiophene, thienothiophene) and acceptor linkers—have emerged as another effective design, where optimized charge separation and exciton dissociation translate into markedly improved photocatalytic CO_2_‐to‐CO performance.^[^ ^158^ ^]^
- A promising strategy involves the incorporation of atomically precise NCs as catalytically active nodes embedded within MOF or COF scaffolds. This nanocluster–framework integration leverages the unique quantum‐size effects and well‐defined active sites of NCs alongside the structural regularity, adjustable pore environment, and host–guest capabilities of reticular frameworks. The framework not only mitigates cluster aggregation and provides spatial isolation to prevent deactivation but also mediates substrate diffusion and product selectivity. Such systems allow for deliberate modulation of both the active site environment and long‐range electronic structure, making them pivotal for complex multielectron CO_2_ reduction reactions.
- A strategic approach involves integrating automated, closed‐loop workflows that connect DFT‐based computational predictions with high‐throughput experimental screening to efficiently interrogate structure–property–function landscapes. By leveraging ML models trained on DFT‐derived descriptors (e.g., binding energies, band positions, charge transfer characteristics), researchers can predict and prioritize promising candidates. These predictions can then be experimentally validated using combinatorial libraries or robotic synthesis platforms, feeding new data back into the model. This iterative design cycle accelerates the discovery of high‐performance catalysts with optimized activity, selectivity, and stability for CO_2_ reduction.

**Figure 11:**
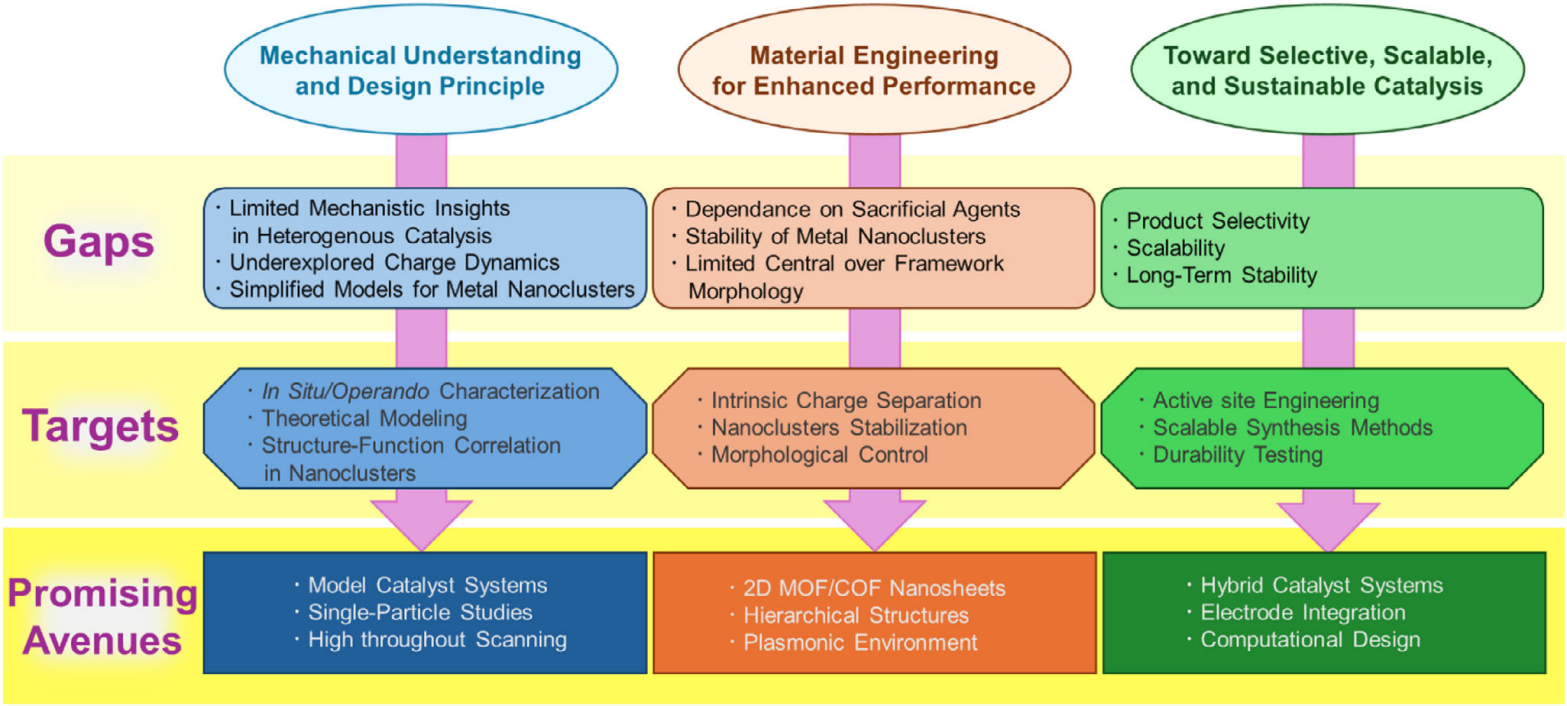
An integrated depiction of the unmet needs, goals, and prospective research trajectories.

An overview of existing gaps, strategic targets, and forward‐looking prospects in this field is concisely illustrated in Figure 11.

## 6 Conclusion

Looking ahead, both metal NCs and reticular frameworks have proven to be indispensable yet intrinsically distinct classes of materials in the advancement of CO_2_RR catalysis. Metal NCs, with their size‐dependent reactivity and tunable electronic configurations, offer a molecular‐level handle to steer reaction selectivity and energy efficiency, particularly for single‐carbon products. Meanwhile, the emergence of MOFs and COFs has opened new avenues for extended periodicity, molecular‐level programmability, and opportunities for multifunctional integration—features that are especially relevant for facilitating multistep electron transfer, co‐reactant diffusion, and selective C─C coupling.

Yet, material‐specific drawbacks remain a barrier. NCs, while highly active, are prone to instability due to their high surface energy, leading to agglomeration and loss of active surface area. Conversely, the extended conjugation and structural modularity of MOFs/COFs are often offset by suboptimal conductivity and operational lifespans. Achieving high selectivity toward C_2+_ products, especially beyond copper‐based systems, remains a formidable hurdle.

Moving forward, hybrid strategies that combine the atomic precision of NCs with the robustness and tunability of extended frameworks may offer synergistic advantages, enabling fine‐tuned control over both the local electronic structure and long‐range transport phenomena. Several promising integration pathways are emerging: i) covalent anchoring of NCs onto functionalized linkers or nodes within MOFs/COFs, which provides strong binding interactions to suppress sintering and surface reconstruction under catalytic turnover^[^ ^159^ ^]^; ii) encapsulation of NCs into well‐defined framework cavities or channels, where steric confinement not only mitigates aggregation but also tailors substrate accessibility and local reaction environments^[^ ^153^ ^]^; iii) post‐synthetic ion‐exchange or cluster‐insertion approaches, in which preformed NCs are selectively introduced into open coordination sites of MOFs/COFs, thereby combining structural periodicity with atomically precise catalytic centers^[^ ^160^ ^]^; and iv) in situ growth of NCs within framework pores or defect sites, a bottom‐up strategy that ensures intimate contact between NCs and framework backbones, often enhancing electron transfer and stability.^[^ ^161^ ^]^ These routes collectively provide a modular toolbox for constructing NC–framework hybrids that exploit both short‐range electronic effects of clusters and long‐range charge/mass transport advantages of porous scaffolds, offering a path toward more durable and selective CO_2_RR catalysts. Besides, the integration of metal active sites within covalently bonded frameworks (MCOFs) offers a synergistic platform that combines catalytic precision with structural durability, positioning MCOFs as a unique hybrid framework poised to address mechanistic and performance bottlenecks in CO_2_ photo‐ and electroreduction. Ultimately, progress in this area will depend not only on materials discovery but also on the convergence of computational modeling, accelerated synthesis platforms, and *operando* spectroscopic tools—together forming a feedback‐rich pipeline to bridge the divide between conceptual design and real‐world performance.

## Conflict of Interests

The authors declare no conflict of interest.

## Data Availability

The data that support the findings of this study are available from the corresponding author upon reasonable request.
